# Molecular Framework of a Regulatory Circuit Initiating Two-Dimensional Spatial Patterning of Stomatal Lineage

**DOI:** 10.1371/journal.pgen.1005374

**Published:** 2015-07-23

**Authors:** Robin J. Horst, Hironori Fujita, Jin Suk Lee, Amanda L. Rychel, Jacqueline M. Garrick, Masayoshi Kawaguchi, Kylee M. Peterson, Keiko U. Torii

**Affiliations:** 1 Howard Hughes Medical Institute, University of Washington, Seattle, Washington, United States of America; 2 Department of Biology, University of Washington, Seattle, Washington, United States of America; 3 National Institute for Basic Biology, Okazaki, Aichi, Japan; Wageningen University, NETHERLANDS

## Abstract

Stomata, valves on the plant epidermis, are critical for plant growth and survival, and the presence of stomata impacts the global water and carbon cycle. Although transcription factors and cell-cell signaling components regulating stomatal development have been identified, it remains unclear as to how their regulatory interactions are translated into two-dimensional patterns of stomatal initial cells. Using molecular genetics, imaging, and mathematical simulation, we report a regulatory circuit that initiates the stomatal cell-lineage. The circuit includes a positive feedback loop constituting self-activation of SCREAMs that requires SPEECHLESS. This transcription factor module directly binds to the promoters and activates a secreted signal, EPIDERMAL PATTERNING FACTOR2, and the receptor modifier TOO MANY MOUTHS, while the receptor ERECTA lies outside of this module. This in turn inhibits SPCH, and hence SCRMs, thus constituting a negative feedback loop. Our mathematical model accurately predicts all known stomatal phenotypes with the inclusion of two additional components to the circuit: an EPF2-independent negative-feedback loop and a signal that lies outside of the SPCH•SCRM module. Our work reveals the intricate molecular framework governing self-organizing two-dimensional patterning in the plant epidermis.

## Author Summary

Generation of self-organized, functional tissue patterns is critical for development and regeneration in multicellular organisms. Small valves on the epidermis of land plants, called stomata, mediate gas-exchange while minimizing water loss. Density and spacing of stomata are regulated by transcription factors that drive differentiation as well as by cell-cell signaling components that regulate entry and spacing of stomatal lineage cells. To unravel how interaction of these components translates into two-dimensional patterning of stomata, we have taken an integrative approach employing molecular genetics, imaging, and mathematical modeling. In this paper we have identified a regulatory circuit controlling the initiation of the stomatal cell lineage. The key elements of the circuit are a positive feedback loop constituting self-activation of the transcription factors SCREAM / SCREAM2 (SCRMs) that requires SPEECHLESS (SPCH), and a negative feedback loop involving the signaling ligand EPF2, the receptor modifier TOO MANY MOUTHS, and the SPCH•SCRMs module. The receptor ERECTA, on the other hand, lies outside of the regulatory loop. Our mathematical modeling recapitulated all known stomatal phenotypes with the addition of two regulatory nodes. This work highlights the molecular framework of a self-organizing patterning system in plants.

## Introduction

Multicellular organisms produce complex tissues, each comprised of specialized cell types with appropriate spatial configuration for optimal function, thus contributing to the fitness of the organism. Seemingly uniform precursor cells self-organize into distinct, functional patterns. A fundamental question to developmental biology is how these patterns are generated through regulatory networks. Stomata are microscopic pores on the plant epidermis surrounded by paired guard cells that can adjust their aperture to mediate efficient gas exchange for photosynthesis while minimizing water loss. Because stomata form in response to spatial cues and cell migration is absent in plants, stomatal patterning is an excellent model to study how local cell-cell interactions create two-dimensional spatial patterns during development.

Over the years, several key components that govern stomatal patterning and differentiation have been identified in Arabidopsis. Stomatal differentiation is directed by the sequential action of basic-helix-loop-helix (bHLH) transcription factors SPEECHLESS (SPCH), MUTE, and FAMA, and their heterodimeric partners SCREAM (SCRM), also known as ICE1, and SCRM2 [1–4]. Inhibitory cell-cell signaling pathways restrict initiation and enforce spacing of stomata. The upstream signaling components are secreted cysteine-rich peptides, EPIDERMAL PATTERNIG FACTOR1 (EPF1) and EPF2, which are perceived by the cell-surface receptors of the ERECTA (ER)-family receptor kinases and the modulator TOO MANY MOUTHS (TMM) [5–9]. The signals are transduced via Mitogen Activated Protein Kinase (MAPK) cascades [10,11]. The MAPKs phosphorylate SPCH to restrict its activity, directly connecting the upstream signaling pathway to a downstream transcription factor [12]. Two paralogs of ERECTA, ERECTA-LIKE1 (ERL1) and ERL2, are expressed in the later steps of stomatal development and restrict asymmetric spacing divisions as well as differentiation of guard mother cells to stomata [5]. This later step is mediated by EPF1, a secreted peptide related to EPF2 [6,9].

Although a lot is known about the signaling pathways and transcription factors controlling stomatal development, it still remains unclear how regulatory interactions of these components will cohesively translate to organized patterns of stomatal-lineage initials from undifferentiated protodermal cells. The initiation of the stomatal cell lineage, i.e. the specification of meristemoid mother cells (MMC) that facilitates entry into asymmetric divisions to create stomatal transient precursors known as meristemoids, is specified by SPCH and SCRMs while being restricted by EPF2, ERECTA, and TMM. Here we use both empirical and modeling approaches to delineate the order of gene product actions in order to deduce the regulatory circuit initiating stomatal-lineage patterns. Our work defines a minimal regulatory circuit comprised of four essential components required that are sufficient to recapitulate observed stomatal patterns: (i) a positive feedback loop mediated at the node of SCRM, with SCRM as a direct target and heterodimeric partner of SPCH; (ii) an EPF2-dependent negative feedback loop inhibiting SPCH•SCRM heterodimers; (iii) an EPF2-independent negative feedback loop inhibiting SPCH•SCRM heterodimers; and (iv) an antagonistic signal competing with EPF2 that is not regulated by the SPCH•SCRM module. Our study reveals the core regulatory framework governing stomatal initiation, as an example to better understand two-dimensional spatial patterning that was proposed nearly three decades ago.

## Results

### The positive-feedback circuit initiating stomatal cell lineages

**Fig 1:**
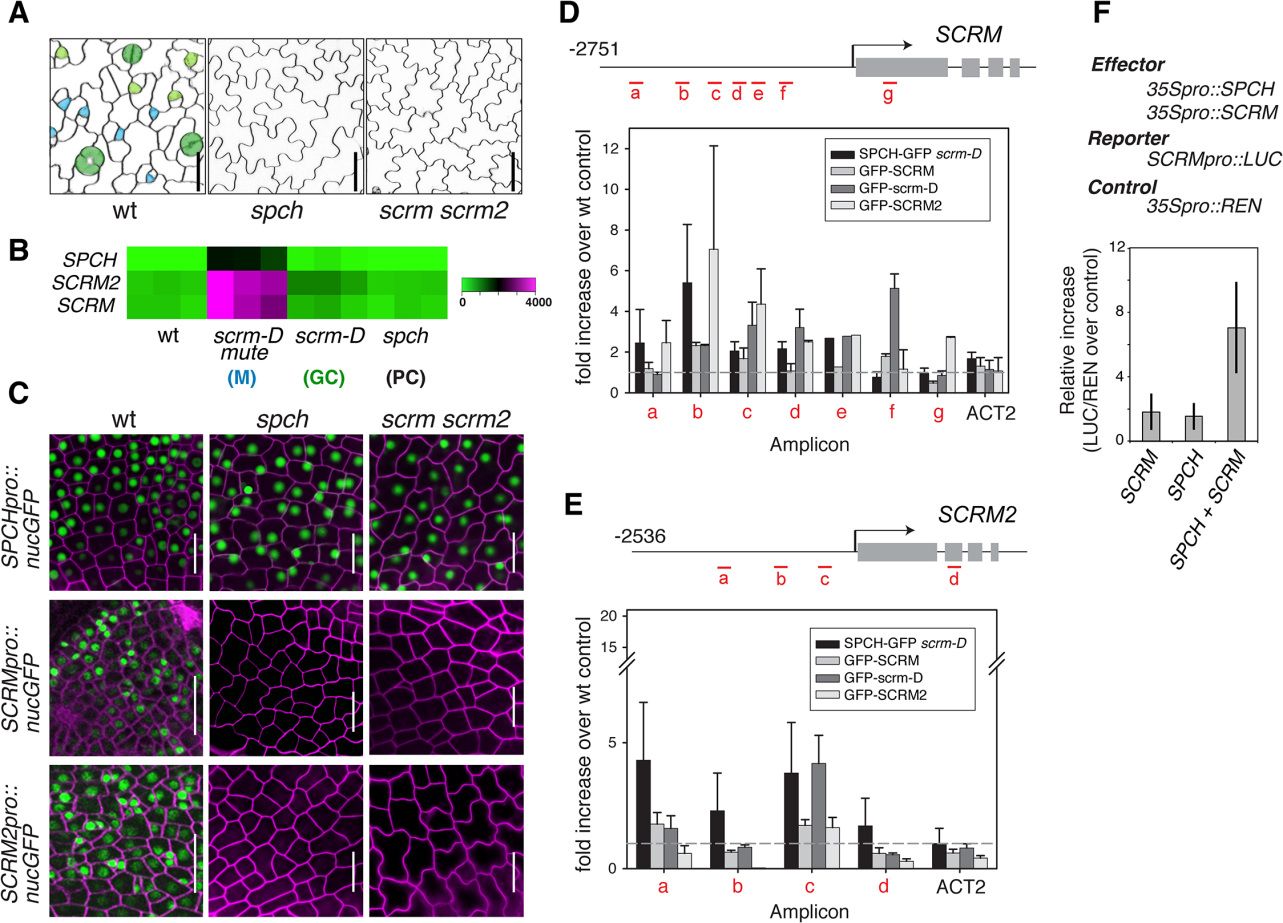
Molecular framework of the SPCH•SCRM positive feedback for stomatal-lineage specification. (**A**) *SPCH* and *SCRM* are mutually required for initiating the entry asymmetric division of stomatal cell lineages. Shown are false-colored confocal microscopy images of abaxial rosette leaf epidermis from 10–12 day-old seedlings. Wild type (left) epidermis gives rise to stomatal lineage cells: Cyan, early meristemoids; light green, late meristemoids and guard mother cells; green, immature and mature guard cells; white, stomatal-lineage ground cells or pavement cells. *spch* or *scrm scrm2* mutant epidermis is solely composed of pavement cells (white). Scale bars, 20 μm. (**B**) Expression heat map of *SPCH*, *SCRM*, and *SCMR2* from a microarray study [13] in wild type and mutants enriched in specific epidermal cells: *scrm-D mute* (M: meristemoids); *scrm-D* (GC: stomatal guard cells); *spch* (PC: pavement cells). (**C**) Promoter GFP reporter expression patterns of *SPCHpro*::*nucGFP* (top), *SCRMpro*::*nucGFP* (middle), and *SCRM2pro*::*nucGFP* (bottom) in early protoderm of 11-day-old wild-type (left), *spch* (middle), and *scrm scrm2* (right) seedlings. *SPCH* does not require itself or *SCRMs* for its own promoter activity. In contrast, *SCRMs* require SPCH and themselves, indicating that SCRMs form a positive feedback loop essential for pattern formation. Scale bar, 20 μm. (**D, E**) ChIP assays on *SCRM* (**D**) and *SCRM2* (**E**) promoter regions using anti-GFP antibody on control wild type or transgenic seedlings expressing functional SPCH-GFP in *scrm-D*, GFP-SCRM, GFP-scrm-D, or GFP-SCRM2. Each amplicon is indicated in a red letter. Shown as a graph are mean ± SEM of fold enrichment over wild-type Col from three biological replicates. Line, intergenic region or intron; arrow, transcription start site; filled rectangle, coding region. (**F**) Transactivation assays in *N*. *benthamiana*. Reporter luciferase expression driven by *SCRM* promoter is strongly induced when both SPCH and SCRM are present. Reporter firefly luciferase activity was normalized against constitutively expressed *Renilla* luciferase, and the values are normalized against controls without effector proteins. Bars indicate means of three biological replicates; error bars, S.E.M.

Phenotypically, loss-of-function *spch* and *scrm scrm2* Arabidopsis seedlings are identical; both develop an epidermis devoid of stomatal cell lineages and thus solely composed of pavement cells. These highly cuticulated and crenulated cells protect internal tissues from desiccation and other environmental stresses (Fig 1A)[4]. Further, *SPCH*, *SCRM*, and *SCRM2* transcripts accumulate in a very similar manner within stomatal cell lineages, with strong enrichment in seedlings that produce an epidermis primarily composed of meristemoids (*scrm-D mute*)(Fig 1B)[13]. To decipher the regulatory relationships between SPCH and two SCRMs, we first examined their transcriptional reporters, *SPCHpro*::*nucGFP*, *SCRMpro*::*nucGFP*, and *SCRM2pro*::*nucGFP*. All reporters are uniformly active in the early protoderm of wild-type leaf primordia (Fig 1C). *SPCH* promoter was active regardless of the presence or absence of functional *SPCH* or *SCRMs* (Fig 1C). In contrast, no GFP signals driven by *SCRM* or *SCRM2* promoters were detected in *spch* or *scrm scrm2* protoderm (Fig 1C), indicating that the expression of *SCRMs* relies on its self-activation as well as *SPCH*.

Unlike the uniform promoter activities, functional GFP fusion proteins of SPCH (*SPCHpro*::*SPCH-GFP*), SCRM (*SCRMpro*::*GFP-SCRM*), and SCRM2 (*SCRM2pro*::*GFP-SCRM2*) accumulated in the nuclei of a subset of protodermal cells and early stomatal precursors (S1 Fig), emphasizing the role of post-transcriptional regulation in the proper establishment of stomatal-lineage cells. Similar to *spch* [14], none of the *scrm scrm2* protodermal cells divide asymmetrically but instead undergo symmetric division (S1 Fig). These cells transiently express SPCH-GFP protein (S1 Fig). In contrast, no GFP-SCRM and GFP-SCRM2 was detected in the *spch* protoderm (S1 Fig). Thus, while SPCH protein could accumulate transiently in the absence of *SCRMs*, SPCH requires its heterodimeric partners (SCRMs) to initiate stomatal cell-lineages.

To address whether *SCRMs* are directly regulated by SPCH and SCRMs, direct binding of these transcription factors to the promoters of SCRM and SCRM2 were tested using chromatin immunoprecipitation (ChIP) assays (Fig 1D and 1E and S2 Fig). For this purpose, we used both the wild-type background and *scrm-D*. The *scrm-D* allele carries an amino-acid substitution within a region of unknown function currently named as the ‘KRAAM’ motif, given the high sequence conservation of this motif amongst land plants [15]. *scrm-D* confers a stomata-only epidermal phenotype [4]. The *scrm-D* mutant serves as an excellent tool to enrich the number of stomatal precursor cells that have been shown to properly express key stomatal lineage genes [4,13]. Association of SPCH-GFP, GFP-SCRM, and GFP-SCRM2 was detected within the 5’ proximal region of the 2.5 kb *SCRM* promoter (Fig 1D). Consistent with the increased numbers of stomatal precursors, GFP-scrm-D ChIP yielded higher signal intensity while binding patterns across the promoter region remained the same as the GFP-SCRM ChIP (Fig 1D and S2 Fig). Similarly, associations of GFP-SCRM, GFP-SCRM2, and SPCH-GFP (in *scrm-D*) were detected in the *SCRM2* promoter (Fig 1E). Transactivation assays using *N*. *benthamiana* showed that both SPCH and SCRM proteins are required to activate *SCRM* reporter expression (Fig 1F). Together, these results place *SPCH* most upstream of a regulatory circuit, where it induces its partners, *SCRMs*, via direct binding to their promoter regions. Furthermore, the results indicate that self-activation of *SCRMs* via direct binding to their own promoters constitutes the molecular basis of a positive feedback loop for robust specification of stomatal-lineage fate.

### The negative-feedback loop restricting the initiation of stomatal cell lineages

**Fig 2:**
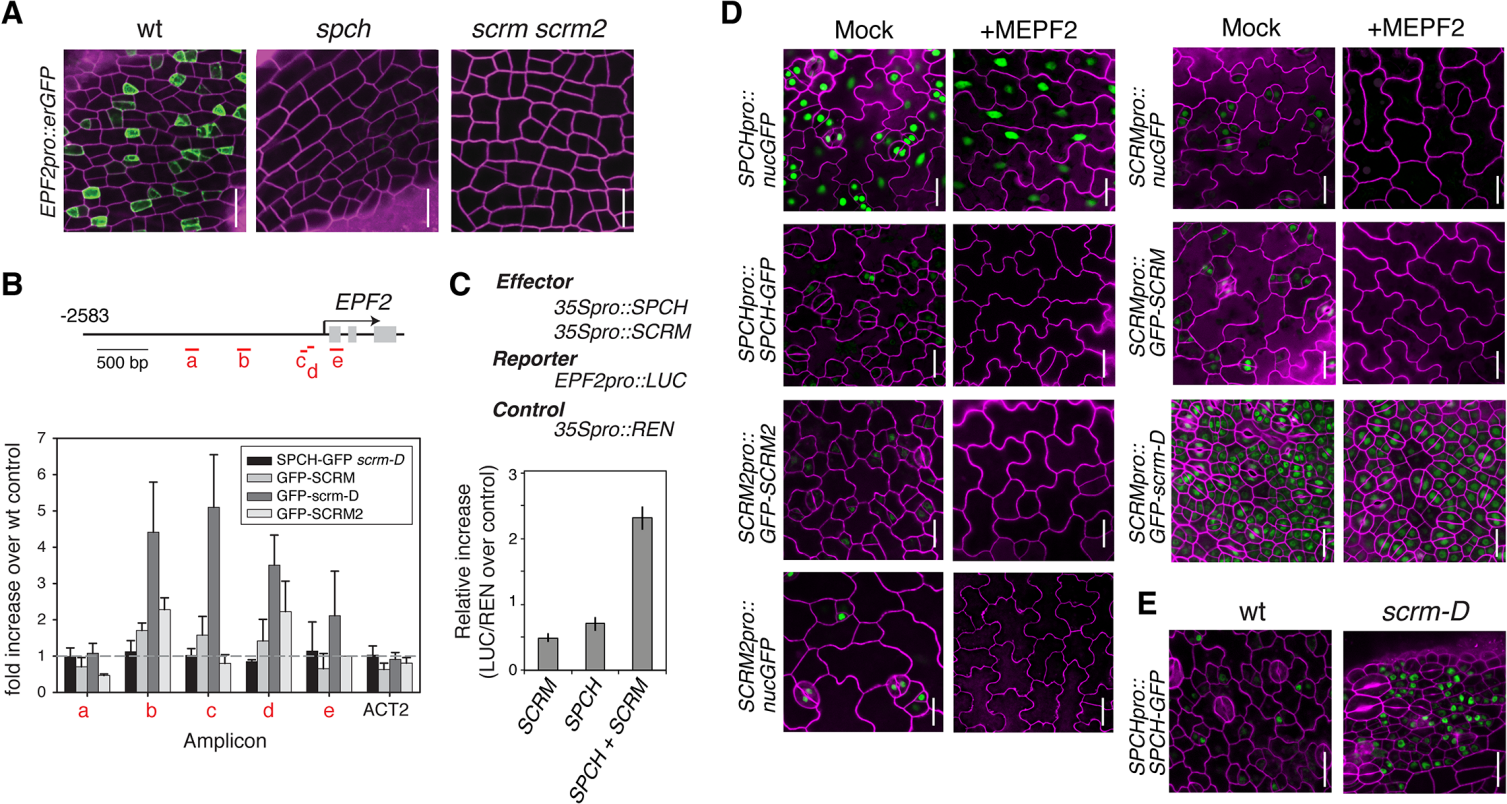
Molecular framework of the negative-feedback loop between SPCH•SCRM and EPF2 for stomatal-lineage specification. (**A**) Shown are confocal images of abaxial protoderm of rosette leaf primordia of 10-11-day-old seedlings expressing *EPF2pro*::*erGFP* in wild type (left), *spch* (middle), and *scrm scrm2* (right). No *EPF2* promoter activity is detected in the absence of *SPCH* or *SCRMs*. Scale bars, 20 μm. (**B**) ChIP assays on *EPF2* promoter region using anti-GFP antibody on control Col or transgenic seedlings expressing functional SPCH-GFP in *scrm-D*, GFP-SCRM, GFP-scrm-D, or GFP-SCRM2. Each amplicon is indicated in a red letter. Mean ± SEM of fold enrichment over wild-type Col from three biological replicates are shown. *ACT2* serves a control. Line, intergenic region or intron; arrow, transcription start site; filled rectangle, coding region. (**C**) Transactivation dual luciferase reporter assays in *N*. *benthamiana*. Strong *EPF2* reporter expression is detected when both SPCH and SCRM are present. Bars indicate means of biological triplicates; error bars, S.E.M. (**D**) Effects of bioactive recombinant MEPF2 peptide application on promoter activity and protein accumulation of SPCH and SCRMs. MEPF2 application has no effect on *SPCH* promoter activity (*SPCHpro*::*nucGFP*) despite the fact that no-stomatal cell linages are initiated (top left). In contrast, MEPF2 application results in loss of GFP signals in *SPCHpro*::*SPCH-GFP* (top right), *SCRMpro*::*nucGFP* (middle left), *SCRMpro*::*GFP-SCRM* (middle right), and *SCRM2pro*::*GFP-SCRM2* (bottom left). GFP-scrm-D protein is insensitive to MEPF2 application (bottom right). Six-day-old cotyledons are imaged under the same magnification. Scale bar, 20 μm. (**E**) Abaxial epidermis from 5-6-day-old seedling rosette leaf primordia expressing *SPCHpro*::*SPCH-GFP* in wild-type (left) or *scrm-D* (right) background, showing that more protodermal cells accumulate SPCH-GFP protein (green) in *scrm-D*. Scale bar, 20 μm.

Stomatal patterning requires negative regulators that ensure proper stomatal spacing and distribution [7,8]. *epf2* loss-of-function confers excessive entry into the stomatal-cell lineage, and conversely, *EPF2* overexpression results in a pavement-cell-only epidermis, a phenotype identical to *spch* or *scrm scrm2* [7–9]. These observations have led to a hypothesis that EPF2-SPCH•SCRMs constitute a negative feedback loop restricting the number of MMCs [7,8,16,17]. No *EPF2pro*::*erGFP* signal was detected in the protoderm of *spch* [7] or *scrm scrm2* (Fig 2A), indicating that both SPCH and SCRMs are required for *EPF2* expression. Subsequent ChIP analysis revealed binding of GFP-SCRM, GFP-scrm-D, and GFP-SCRM2 to the *EPF2* promoter region (Fig 2B and S2 Fig). While association of SPCH with the *EPF2* promoter was not clearly detected, SPCH and SCRM together were capable of transactivating EPF2 reporter expression *in planta* (Fig 2C). This suggests that SPCH and SCRM together induce *EPF2* gene expression.

We next addressed whether EPF2-mediated inhibitory signals target SPCH and/or SCRMs *in vivo*. As reported [9], application of mature EPF2 peptide (MEPF2; 1 μM) completely blocks the initiation and progression of stomatal-cell linages (Fig 2D). Under such conditions, however, strong *SPCHpro*::*nucGFP* signals were uniformly detected in the epidermis, indicating that MEPF2 has no effect on *SPCH* promoter activity (Fig 2D). In contrast, SPCH-GFP protein was not detected after MEPF2 application (Fig 2D). Because *SCRMs* are direct targets of SPCH (Fig 1), their transcription would not occur in the absence of SPCH protein accumulation. Consistently, neither promoter activities nor protein accumulation of SCRM and SCRM2 were detected after MEPF2 application (Fig 2D).

MEPF2 application showed no effects on the stomata-only phenotype and GFP accumulation of *SCRMpro*::*GFP-scrm-D* seedlings (Fig 2D). Thus scrm-D protein is resistant to MEPF2-mediated inhibition. Given that both SPCH and SCRMs must be present to initiate stomatal differentiation, we further examined SPCH protein accumulation in the *scrm-D* background. Indeed, strong SPCH-GFP signals are detected in the *scrm-D* protoderm (Fig 2E). Combined, our results molecularly define the two key nodes of the EPF2-SPCH•SCRM negative feedback loop: the direct regulation of *EPF2* gene expression by SCRMs; and targeted destabilization of SPCH by EPF2-mediated signaling, which can be avoided in the presence of SPCH’s stabilizing partner, scrm-D (Fig 2E).

### Signaling receptors within and outside of the regulatory loop

During leaf development, EPF2 is primarily perceived by ERECTA, which forms homodimers as well as heterodimers with TMM [9]. Although the direct ligand-receptor binding and receptor dimerization have been established biochemically, it is unclear how these two receptors fit into the regulatory circuit initiating stomatal-lineage cells. To address this, we examined the expression and regulation of these receptors.

**Fig 3:**
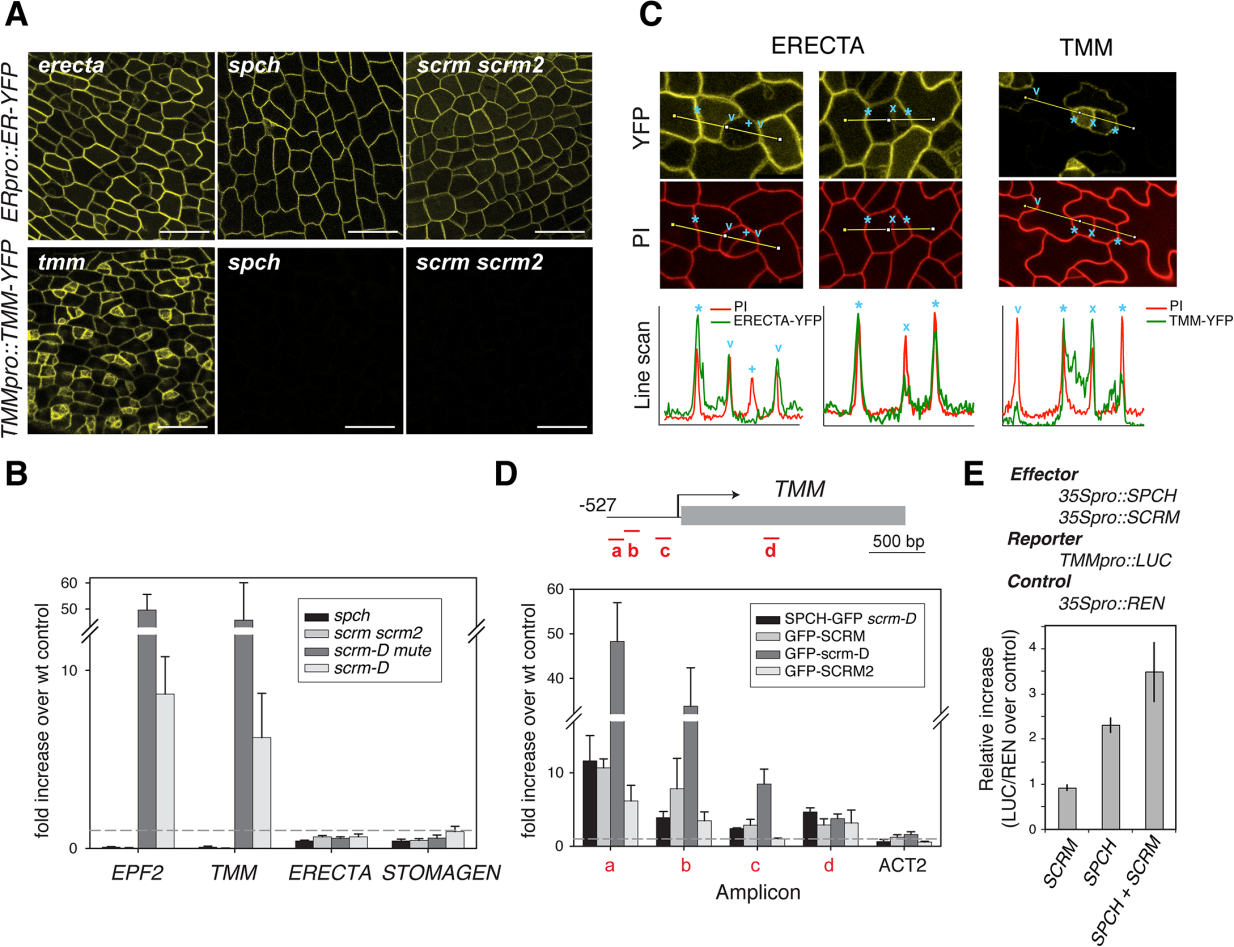
Differential regulation of receptors by SPCH•SCRM module. (**A**) Expression/accumulation patterns of functional ERECTA-YFP (top) and TMM-YFP (bottom) in protoderm from first rosette leaf primordia of 5-8-day-old *erecta tmm* (left), *spch* (middle), and *scrm scrm2* (right) seedlings. No TMM-YFP signal can be detected in the absence of *SPCH* or *SCRMs*. Scale bars, 150 μm. (**B**) qRT-PCR analysis of *EPF2*, *TMM*, *ERECTA*, and *STOMAGEN* transcripts levels from five-day-old *spch* (pavement cells only), *scrm scrm2* (pavement cells only), *scrm-D mute* (meristemoid enriched), and *scrm-D* (stomata enriched) seedlings compared to wild-type. Both *EPF2* and *TMM* transcripts are highly enriched in meristemod-enriched population (*scrm-D mute*) while undetectable in *spch* or *scrm scrm2*. In contrast, *ERECTA* and *STOMAGEN* show no such trends. (**C**) Higher magnifications of protoderm expressing ERECTA-YFP levels (top left and middle) and TMM-YFP (top right) co-stained with PI (middle) to highlight cell periphery. Presented at the bottom are line scan analyses of each panel corresponding to lines indicated in the confocal images. Cell boundaries between a stomatal-lineage cell and an adjacent epidermal cell (asterisks), between a meristemoid and an SLGC (x), between a GC and adjacent epidermal cells (v), and between two paired GCs (+) are indicated. ERECTA-YFP levels are reduced in stomatal precursors and not detectable in GCs, while TMM-YFP levels are stomatal-lineage-specific (**D**) ChIP assays on *TMM* promoter region using anti-GFP antibody on control Col-0 or transgenic seedlings expressing functional SPCH-GFP in *scrm-D*, GFP-SCRM, GFP-scrm-D, or GFP-SCRM2. Each amplicon is indicated by a letter. Shown are the means ± SEM of fold enrichment over wild type Col from three biological replicates. Line, intergenic region or intron; arrow, transcription start site; filled rectangle, coding region. (**E**) Transactivation dual luciferase reporter assays using *N*. *benthamiana*. *TMM* expression is upregulated when both SPCH and SCRM are present. Bars indicate means of triplicate; error bars, S.E.M.

Functional ERECTA-YFP protein driven by its own promoter (*ERECTApro*::*ERECTA-YFP*) showed uniform signals in the plasma membrane of nearly all protodermal cells (Fig 3A). Neither *spch* nor *scrm scrm2* mutation affected ERECTA-YFP signals (Fig 3A). The ERECTA protein accumulation pattern was consistent with its transcript levels (Fig 3B). Therefore, *ERECTA* expression is not dependent on the SPCH•SCRMs module. In the maturing leaf epidermis, ERECTA protein levels (ERECTA-YFP) appear higher in the pavement cells than in stomatal precursors (Fig 3C).

In contrast to ERECTA, no TMM-YFP signals were detected in *spch* or *scrm scrm2* mutant backgrounds (Fig 3A). Consistent with a previous report [18], functional TMM-YFP protein driven by its own promoter (*TMMpro*::*TMM-YFP*) accumulates strongly in MMCs and meristemoids, somewhat less in meristemoid sister cells (stomatal-lineage ground cells, SLGC), and is barely detected in pavement cells (Fig 3A and 3C). *TMM* transcript levels across stomatal cell-state mutants [13] accord with the observed TMM-YFP signals, and *TMM* shows very similar expression trends to *EPF2* (Fig 3B). The ChIP assays within the established 527 bp *TMM* promoter, which fully rescues *tmm* mutant phenotypes [5,18] detected binding of SPCH-GFP, GFP-SCRM, and GFP-SCRM2 (Fig 3D and S2 Fig). GFP-scrm-D significantly enhanced the signal without altering the binding patterns (Fig 3D and S2 Fig). Further, a dual-luciferase transactivation assay showed robust induction of *TMM* promoter activity *in planta* in the presence of both SPCH and SCRM (Fig 3E). Taken together, our results highlight the contrasting expression pattern and regulation of two EPF2 receptors: ERECTA, the main receptor situated outside of the SPCH•SCRMs regulon, and TMM, the signal modulator, activated by SPCH•SCRMs.

### Modeling regulatory circuit behavior

We have experimentally deciphered the regulatory architecture of stomatal initiation pathways, which resembles the reaction-diffusion (RD) system presenting Turing-like stabilities. Such a system is capable of self-generating complex and dynamic patterns despite the minimal components involved: i.e. presence of an activator and an inhibitor [19–21]. The less diffusive activator, which in this case corresponds to the SPCH•SCRM module, must activate itself by forming a positive feedback loop (Fig 1). The activator also induces a highly diffusive inhibitor, in our case the secreted peptide EPF2, which in turn inhibits the activator, forming a negative-feedback loop (Fig 2). However, the circuitry is by no means this simplistic. For instance, the EPF2 receptor ERECTA can form both homodimers and heterodimers with TMM *in vivo* [9], and the expression of ERECTA and TMM is regulated differently (Fig 3).

We constructed a computational model to test whether the regulatory circuit unveiled in this study is sufficient to generate two-dimensional spatial patterning capable of initiating the stomatal cell lineage at single-cell resolution. Our intention here is to deduce a minimal set of components that is sufficient to recapitulate patterning of stomatal initial cells. We initially tested whether the core Turing model comprising of activators and inhibitors, in this case SPCH•SCRM module and EPF2-mediated pathway, and their experimentally verified regulatory relationships could explain the patterns of stomatal initial cells. For this purpose we defined a series of ordinary differential equations to describe the circuit (S1 Text). Based on the experimental data, the system has been described as the following: (i) *SPCH* promoter is uniformly active; (ii) SPCH and SCRMs form a heterodimer, which activates *SCRMs* expression (positive feedback); (iii) SPCH•SCRM heterodimer activates *EPF2* and *TMM*; (iv) EPF2-ERECTA/TMM signal leads to the degradation of SPCH and SCRM (negative feedback); and (v) scrm-D is resistant to EPF2-mediated inhibition. Our modeling simplifies the unequal redundancy among three ERECTA-family RKs [5] and sets the diffusion rate of EPF2 far greater than that of the nuclear-localized SPCH and SCRMs. The regulatory circuit consists of both a signaling cascade, in which protein phosphorylation immediately relays signals, and a gene expression cascade, where transcription and then translation would take place The model includes a time-lag in SPCH/SCRM-regulation of TMM and EPF2 expression, while EPF2 perception of receptors to MAPK activation occurs immediately, in successive steps to reflect the time lag (see S1 Text). Each two-dimensional lattice of 400 hexagons represents a sheet of the protoderm with initial state, where each component is introduced with 10.0% random noise. Accumulation patterns of each of the components were analyzed (see S1 Text).

The simulation demonstrated evenly spaced peaks of high SPCH, SCRM, EPF2, and TMM protein levels in single cells, representing stomatal initials, with EPF2 diffusing to neighboring cells (S3 Fig). The initial simulation reproduced *spch*, *scrm scrm2*, and *scrm-D* mutant phenotypes (S3C Fig). However, it failed to reproduce two phenotypes: (i) *erecta* (*erecta*-family) mutant phenotype: The *erecta*-family mutants differ from *scrm-D* in that they develop an epidermis with clustered stomata that align like a chain, surrounding non-stomatal pavement cells [5] (see also S4 Fig). This phenotype can be traced back to the early protoderm, where MMCs accumulating GFP-SCRM form clusters (S3B Fig). However, in our simulation, the *erecta*-family mutants produced an epidermis solely composed of stomatal precursors, just like *scrm-D* (S3C Fig); (ii) Effects of MEPF2 application to *tmm* mutants: MEPF2 application does not suppress the stomatal cluster phenotype of *tmm* (S5B Fig). However, in our model, *tmm* phenotype became suppressed (S3D Fig). This indicates that our initial circuit was incomplete.

### Additional negative feedback in self-organized patterning of stomatal initials

**Fig 4:**
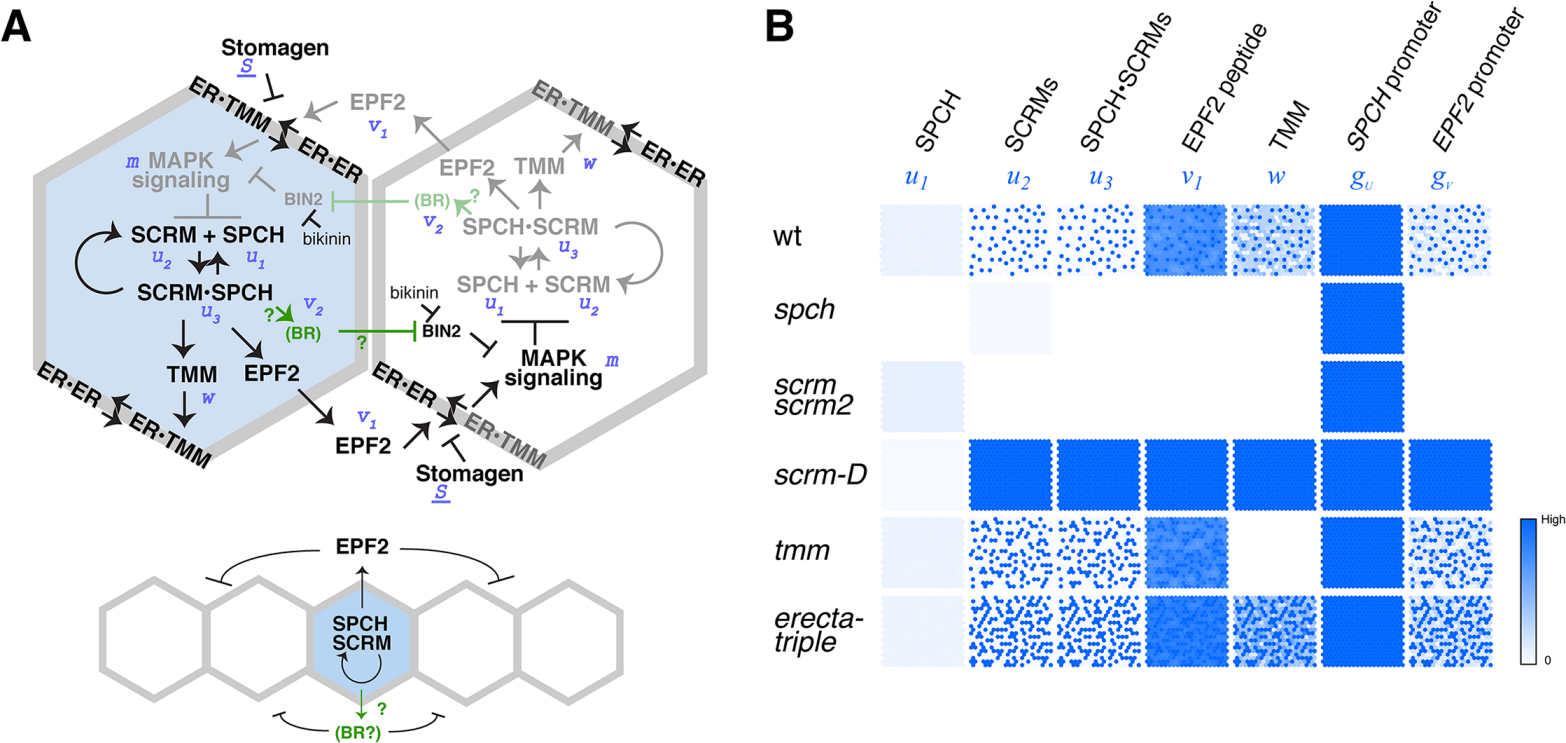
Regulatory circuit modeling two-dimensional patterns of stomatal initial cells. (**A**) Diagram outlining the regulatory circuit used for modeling. (Top) Example of two adjacent protodermal cells undergoing fate determination process. Arrow designates activation and T-bar designates inhibition. Concentrations of each components are abbreviated as the following: *u*_1_, SPCH; *u*_2_, SCRM; *u*_3_, SPCH•SCRM heterodimer; *v*_1_, EPF2; *w*, TMM; *v*_2_, EPF2-independent hypothetical component, most likely BR pathway; *m*, strength of MAPK cascade-mediated inhibition. *S*, a component that competes for receptor pools, most likely Stomagen. The site of bikinin action is also indicated. Initially, all cells possess and operate identical regulatory circuit. Stochastic noise will be amplified in such a way that a cell expressing more activator will self-activate its stomatal-lineage character (light blue), while the neighboring cell will lose stomatal-lineage character (white). The regulatory relationships that are not experimentally verified are in green. It is not known which protodermal cells produce BR, or whether BR acts in neighboring cells. (Bottom) Simplified diagram showing the putative range of inhibitor action. (**B**) Spatial patterns of each component in wild-type and each mutant background simulated *in silico* based on the mathematical models. Each square represents a sheet of protoderm with 400 cells (each cell represented by a hexagon). White cells indicate no expression/accumulation of a given component, while dark-blue cells express/accumulate high amounts.

**Fig 5:**
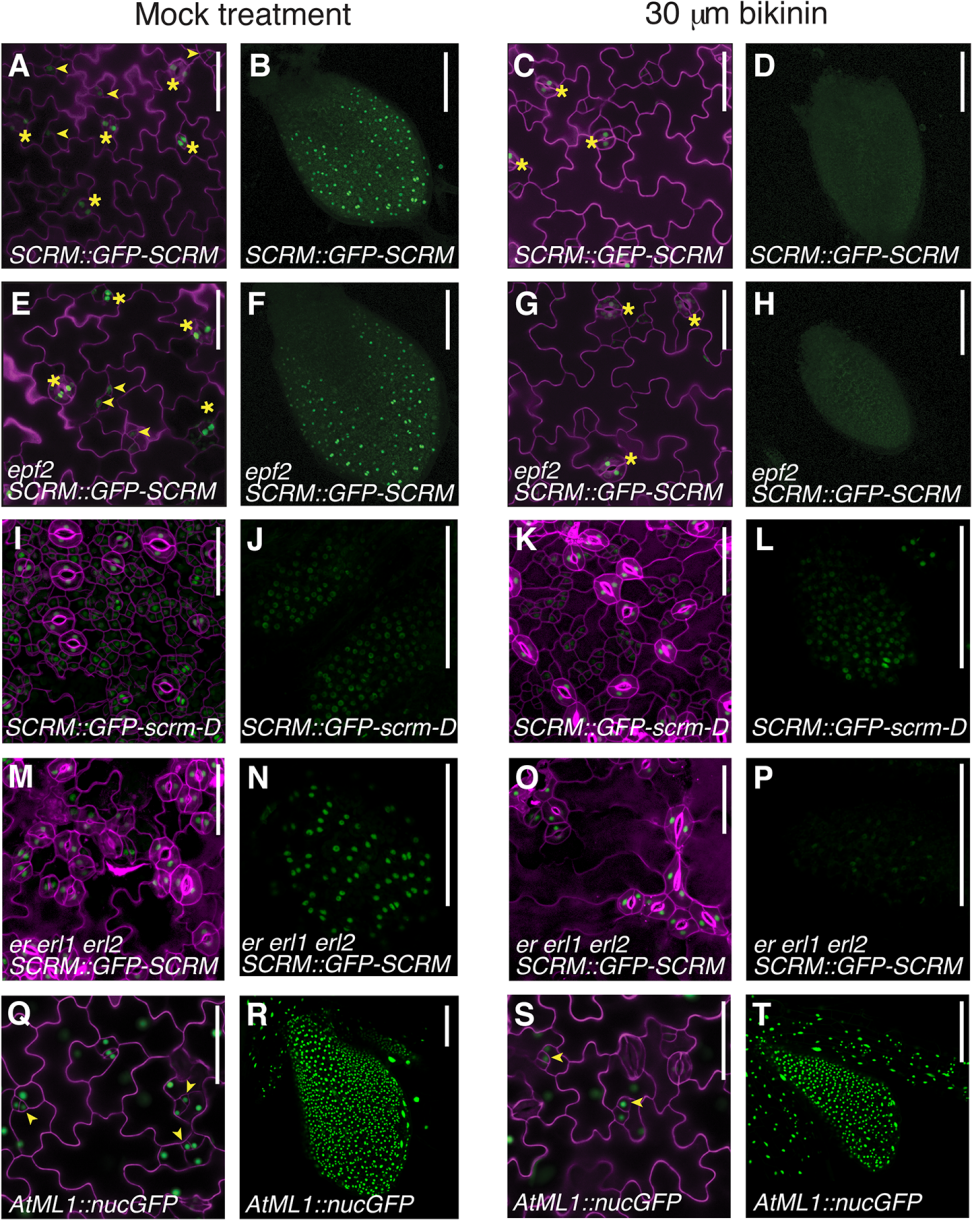
Bikinin treatment represses GFP-SCRM accumulation independent of EPF2-and ERECTA-family. The bikinin-sensitive, EPF2-independent pathway may constitute the second feedback loop predicted by our modeling. (**A-D**) wild-type seedlings carrying *SCRM*::*GFP-SCRM* mock treated (A, B) or treated with 30 μM bikinin (C, D). (**E-H**) *epf2* seedlings carrying *SCRM*::*GFP-SCRM* mock treated (E, F) or treated with 30 μM bikinin (G, H). (**I-L**) wild-type seedlings carrying *SCRM*::*GFP-scrm-D* mock treated (I, J) or treated with 30 μM bikinin (K, L). (**M-P**) *er erl1 erl2* seedling carrying *SCRM*::*GFP-SCRM* mock treated (M, N) or treated with 30 μM bikinin (O, P). (**Q-T**) wild-type seedlings carrying *AtML1*::*nucGFP* mock treated (Q, R) or treated with 30 μM bikinin (S, T). Shown are 5-day-old cotyledon epidermis (A, C, E, G, I, K, M, O, Q, S) and protoderm of primary leaf primordial (B, D, F, H, J, L, N, P, R, T) after 2-day exposure to bikinin. Under bikinin treatment, GFP-SCRM signal disappears from stomatal precursors (arrowheads), while GFP-SCRM in stomata (asterisks) is still detected. Reduction of the GFP-SCRM signal was evident ~ 8 hrs after bikinin treatment and the signals became undetectable 2 days after treatment. For cotyledons, cell periphery was highlighted by propidium iodide; scale bars, 50 μm. For primary leaves, scale bars, 100 μm.

To uncover missing components, we further analyzed the circuit architecture by adding extra regulatory nodes and simulating the outcome. The stomatal clustering phenotype of *erecta*-family mutants can be correctly predicted if we include an additional negative feedback loop that is independent of EPF2, activated by the SPCH•SCRM module, and converging downstream into the ERECTA pathway (Fig 4). Recently, the plant brassinosteroid (BR) hormone-signaling pathway has been shown to influence stomatal development [22,23]. In cotyledons and leaves, an intermediate negative regulator of BR signaling, BIN2, phosphorylates and inhibits the components of the MAPK cascade or directly phosphorylates SPCH itself [22–24]. These reports place the BR pathway as a likely candidate for our mathematically predicted additional negative feedback circuit. To experimentally test this, the effects of bikinin, an inhibitor of BIN2 [22,25], on SCRM protein accumulation, were examined in the presence or absence of *EPF2* or *ERECTA-*family genes (Fig 5). Bikinin treatment reduced GFP-SCRM signals in wild-type, *epf2* and *er erl1 erl2* mutant backgrounds (Fig 5). This is consistent with the phenotypic effects of bikinin on *er erl1 erl2* reported previously, where bikinin treatment rescues the stomatal clustering phenotype to a nearly normal appearance [22] (also see S6 Fig). Specifically, the GFP-SCRM signals in meristemoids disappeared upon bikinin treatment regardless of the presence or absence of *epf2* mutation, while those in mature GCs remained (Fig 5A, 5C, 5E and 5G). In the protoderm of wild-type and *epf2* leaf primordia, no GFP signal was detected after bikinin treatment (Fig 5B, 5D, 5F and 5H). In the absence of *ERECTA-*family, GFP-SCRM was still detected in protodermal cells upon bikinin treatment, but dramatically reduced compared to mock (Fig 5N and 5P). In contrast, accumulation of GFP-scrm-D was only minimally affected by bikinin (Fig 5I–5L). Bikinin treatment did not affect accumulation of nuclear GFP driven by the protodermal promoter *AtML1* [26], indicating that the loss of GFP-SCRM signal is not due to a general toxicity of bikinin (Fig 5Q–5T). These results support the prediction of our modeling that dual negative-feedback loops, one mediated by EPF2-ERECTA and the other independent, integrate into the downstream signaling pathway to inhibit the SPCH•SCRM module.

### Presence of antagonistic ligands outside of the SPCH•SCRM regulons recapitulates the *tmm* phenotype

A loss-of-function *tmm* mutant produces stomatal clusters that are much milder than those of *erecta*-family triple mutant [5,18]. Nevertheless, the *tmm* phenotype is not suppressed by MEPF2 application (S5B Fig). To reconcile this apparent paradox, we introduced additional parameters to our model. Interestingly, including an additional signaling ligand (S) that lies outside of the SPCH•SCRM module enabled *in silico* recapitulation of MEPF2 effects on *tmm* (S5 Fig, S7 Fig and S1 Text). Because the level of S is set constant, increasing amounts of EPF2 reduce the binding of S to the corresponding receptors, ERECTA and TMM. In the *tmm* background, the available excess pool of S to the ERECTA homodimers would counteract MEPF2 application (S5D Fig and S7 Fig). An EPF-LIKE peptide, Stomagen (EPFL9), satisfies all known criteria for S. Stomagen positively regulates stomatal differentiation, acting antagonistically to EPF2 [27–29]. *STOMAGEN* is expressed in undifferentiated mesophyll tissue [27,28], so is unlikely to be regulated by the SPCH•SCRM module. Indeed, *STOMAGEN* transcript accumulation is unaffected by the presence or absence of functional SPCH or SCRMs (Fig 3B).

### Tuning parameters for patterning stomatal initial cells *in silico*

The modified circuit was sufficient to correctly predict all mutant and transgenic phenotypes (Fig 4). Using this minimal circuit enables us to predict the roles of critical parameters that cannot be readily addressed experimentally due to technological limitations and unavailability of appropriate resources. In general, the activator-inhibitor system requires that the diffusion constant for the inhibitors (*d*_*v*_) is substantially larger than that of the activator (*d*_*u*_) in order for patterns to emerge [19,20,30,31]. Furthermore, the ratio of the diffusion constants (*d* = *d*_*v*_/*d*_*u*_) must be larger than the minimal value (*d* > *d*_*min*_), which depends on the model conditions. In this study, *d* = 100.0 was used for the simulation with *G* = 1.0 (*G* is a coefficient for the reaction rate of the negative feedback loop). Under this condition, stomatal patterning was recapitulated robustly (see S1 Text). The exact diffusion rate of EPF2 remains unknown. We therefore tuned our parameter to test what value that constitutes difference between the diffusion rates of the inhibitors and activators would be optimal for predicting the patterns of stomatal initials *in silico*. As shown in S8 Fig, as we reduce the value of *G*, *d*_*min*_ becomes smaller. At *G* = 0.1, our simulation produced stomatal precursor patterning at *d* ≥ 5.0 (S8 Fig). Thus, depending on the rate of negative feedback, a 5-fold difference between the diffusion rates of inhibitors and activators could contribute to the initiation of stomatal patterning.

Next, our assumption of cooperativity (of the Hill function) was investigated *in silico*. The Hill coefficients of *p* = 2 and *q* = 3 were used to model the activation of *SCRM*, *EPF2*, and *TMM* gene expression by SPCH•SCRM module and degradation of the SPCH•SCRM module by MAPK cascade, respectively (see Fig 4A and S1 Text). A simulation was performed to test the effects of cooperativity on the two-dimensional patterning of stomatal initials (S9 Fig). A series of simulations revealed that the values for cooperativity *p* ranging between 1.4 and 2.6 (when *q* = 3.0) were required for spatial patterning of stomatal initials (S9 Fig). Most critically, we were unable to find any conditions that create patterns when no cooperativity in parameter *p* is included (*p* = 1.0). Our results emphasize that the cooperativity for expression of *SCRM*, *EPF2*, and *TMM* by the SPCH•SCRM module is essential for pattern formation of the stomatal initial cells.

In contrast to cooperativity *p*, our simulation showed that robust patterning occurs in any cooperativity for SPCH•SCRM degradation (*q* ≥ 1.0). Even in no cooperativity for *q* (*q* = 1.0), stomatal patterns can be simulated within a narrow range of *p* (S9 Fig). We thus conclude that the cooperativity for SPCH•SCRM degradation may not be absolutely required for patterning. These simulations serve as a guide to investigate the actual biochemical mechanisms of these processes in the future.

## Discussion

This study establishes the molecular framework of a regulatory circuit capable of generating two-dimensional patterning of stomatal cell lineages in the plant leaf epidermis. Evidence from both experimental approaches as well as computational simulations highlight the role of a positive feedback of the SPCH•SCRM module in generating stomatal initials. Direct binding of the transcription factors to promoter regions of cell-cell signaling components, such as *TMM* and *EPF2*, reveals the molecular connection between the positive and negative regulators of stomatal development. The Mature EPF2 peptide in turn inhibits SPCH protein accumulation [9], thereby constituting negative feedback. While stomatal differentiation involves a series of asymmetric cell divisions and cell polarity changes in the later steps of development, which has been modeled [14], our results emphasize that the initial regulatory circuit within the protoderm can generate robust spatial patterns.

The regulatory circuit proposed here predicts that the loss of EPF2 function or ERECTA signaling would phenocopy *SPCH* overexpression. This is indeed the case: the epidermis of *epf2*, dominant-negative *ERECTA* in *er*, and estradiol-inducible *SPCH* overexpression all confer similar phenotypes of enhanced entry into stomatal cell lineages [1,2,7,9] (S7 Fig). None of these genotypes confer constitutive stomatal differentiation as seen in *scrm-D* or *mpk3 mpk6* double mutants [4,11]. This is likely owing to the additional, EPF2-independent negative-feedback loop that merges into the MAPK cascade (see Fig 4). Additionally, there exists a mechanism that restricts stomatal differentiation later in stomatal development, likely mediated by an EPF1-ERL1 signal-receptor module [9]. This scenario is consistent with the fact that stomatal clustering phenotype is only visible in complete loss-of-function in three *ERECTA*-family genes.

We found that two major components of stomatal development, *SPCH* promoter activity and *ERECTA* protein accumulation, are not regulated by the feedback loops. Uniform accumulation of the ERECTA protein in the entire protoderm regardless of the presence or absence of the activators (SPCH and SCRMs) allows rapid signal transduction as the activators induce the diffusible ligand (EPF2). Based on the Reaction-Diffusion hypothesis, EPF2 diffuses much faster than the activators, which are nuclear-localized SPCH and SCRMs, and this allows cells expressing higher amounts of SPCH and SCRM (hence EPF2) to adopt MMC identity while preventing the adjacent neighboring cells to do so. Our simulation (S8 Fig) shows that the difference between the rates of diffusion could be as small as five fold or up to 100 fold, depending on the strength of negative feedback loop. Direct visualization of EPF2 diffusion, while technically challenging, could enable us to constrain this parameter in the future.

The actual biochemical mechanism responsible for the self-inhibition of stomatal initial cells remains unclear. It is interesting to hypothesize that a signal modulator, TMM, may bias the strength of inhibition between an MMC and surrounding neighboring cells. *TMM* expression is directly regulated by the SPCH•SCRMs module and exhibits a nearly identical expression pattern as *EPF2*. This could create different stoichiometry of their receptor homo/heteromers among the epidermal population: a higher TMM: ERECTA ratio in stomatal precursors to buffer inhibitory signals, and a lower TMM: ERECTA ratio in neighboring cells (see Fig 3C) to efficiently discriminate the response. This hypothesis precludes ERECTA-TMM heterodimers as the sole EPF2-signal transducers, and favors the additional role of the ERECTA homodimers or ERECTA receptor complex with other co-receptors in repressing initiation of stomatal cell lineages.

We have reported previously that interactions between *ERECTA*-family genes and *TMM* are highly context- and genotype dependent: All three *ERECTA*-family genes together act antagonistically to *TMM* in the stem and hypocotyl epidermis, whereas *ERL1* acts antagonistically to *TMM* in the cauline leaf and carpel epidermis [5]. Unlike these organs, we did not observe specific effects of *tmm* mutation on the cotyledon epidermis and rosette leaf protoderm of different combinations of *erecta*-family higher-order mutants (S10 and S11 Figs). Thus, while our minimal circuit model can accurately explain the behaviors of TMM and ERECTA-family in the cotyledons and primary leaves, stomatal patterning in other organs likely requires additional regulatory nodes to distinguish unique contributions of each ERECTA-family RKs.

Our minimal circuit model predicts that, in sharp contrast to SPCH, TMM expression levels have rather modest effects in overall numbers and patterning of stomatal initial cells (S12 Fig). This is consistent with the predicted role of TMM in attenuating ERECTA-family signaling [9].

Furthermore, having ERECTA in the protoderm prior to stomatal-lineage initiation may be important for the action of Stomagen, an EPF-LIKE peptide expressed in the mesophyll, to promote stomatal development [27,28]. Like ERECTA, Stomagen is not regulated by SPCH•SCRM (Fig 3B). It is fascinating to predict that Stomagen inhibits ERECTA signaling via direct binding, which in turn enables stable accumulation of SPCH and subsequently induces components of the feedback loop. Our biochemical studies indicate that MEPF2 and Stomagen indeed do compete for binding to ERECTA [32]. The inclusion of a Stomagen-like signal, S, in our mathematical model (S7 Fig) was not necessary for the recapitulation of stomatal initial patterns in wild-type and all mutants simulated, but was indispensable for reproducing the stomatal cluster phenotype of *tmm* upon MEPF2 application (S5 Fig). This highlights the added intricacy in the peptide-receptor system, which may reflect the roles of TMM for buffering multiple EPF/EPFL signals [33–35].

Recently, Lau et al. (2014) reported a genome-wide identification of SPCH-downstream targets by ChIP-sequencing, which also identified *SCRMs* and *TMM* as direct targets [36]. The authors observed SPCH binding to the *EPF2* promoter, between -250 and -900, coinciding with the region of direct SCRM and SCRM2 protein binding (Fig 2B). We were not able to detect SPCH binding to that region, although we did detect binding of SPCH to *SCRM*, *SCRM2*, and *TMM* promoter regions. It is important to note that a site-directed mutagenized, MAPK-resistant variant of SPCH was used by Lau et al. (2014) to enhance signals, whereas wild-type SPCH-GFP in *scrm-D* was used in our study. It is likely that the extra-large sampling scale (MOBE-ChIP) by Lau et al. (2014) can capture weak SPCH binding sites more efficiently. At the same time, it is also possible that SPCH phosphorylation status may influence binding to some target genes more so than to others. It has been shown that the phosphorylation status of animal bHLH proteins, MyoD and E47, influence DNA binding as well as its dimerization dynamics [37,38].

Our simulation further predicted that an additional negative feedback loop independent of EPF2 must act on the SPCH•SCRM module in order to generate proper stomatal patterns (Fig 4). Integration of BR-signaling components into the MAPK cascade downstream of EPF2 satisfies this condition both experimentally [39] and in our modeling efforts, although it does not preclude the presence of additional feedback modules. The dual negative feedback model assumes that SPCH•SCRM activates the BR biosynthesis and/or signaling pathway. Recent, genome-wide ChIP-seq analysis of SPCH-binding sites identified a set of BR-biosynthesis and signaling genes as direct targets of SPCH [36], supporting our model. These genes are upregulated upon SPCH-induction [36]. Whether SCRMs bind to the same promoter regions of these SPCH-regulated BR genes is an interesting future topic.

In contrast to cotyledons and leaves, where BRs restrict stomatal development, BRs promote stomatal development in hypocotyls via preventing BIN2-mediated direct phosphorylation of SPCH [23]. BR effects on hypocotyl stomatal production have been reported to act downstream or independent of EPF2 or the ERECTA-family [23]. Surprisingly, we found that bikinin-treatment triggered complete loss of stomatal development in *erecta*-family triple mutants, a phenotype opposite to that of the one predicted by Gudesblat et al. 2012 (S6B Fig). This suggests that the regulatory relationships between BR and the EPF2-ER modules differ in hypocotyls. Deciphering the organ-specific wiring of this regulatory circuit remains a question of future interest.

Stomata, that serve as the interface between the plant and the atmosphere, are influenced by diverse environmental factors during development. Our mathematical model is robust, and increasing the strengths of random noise did not influence the two-dimensional patterning of stomatal cell lineages (S13 Fig), implying that the environmental input alters the parameters or key regulatory nodes or the circuit architecture to change the outcome. For instance, high CO_2_ concentration induces expression of EPF2 and a protease that cleaves and activates the EPF2 propeptide [40]. Thus, high CO_2_ concentration introduces an additional signal that feeds into the regulatory circuit by activating EPF2 signaling. It would be interesting to address in the future how such additional components influence our minimal circuitry governing core stomatal patterning.

During Arabidopsis rosette leaf epidermal development, an additional cell type, the trichome, also differentiates in an evenly spaced manner [41]. The underlying mechanism of trichome patterning has been investigated both experimentally [42,43] and mathematically [44], and involves the cell-to-cell movement of transcription factors and scaffold proteins via plasmodesmata. This leads to trapping and depletion of transcriptional activators. Therefore, the actual execution of spatial patterning may involve distinct molecular mechanisms. The *scrm-D* mutation triggers constitutive stomatal differentiation at the expense of trichome differentiation [4]. In addition, *TMM* overexpression was recently shown to reduce trichome numbers, indicating cross-talk between trichome and stomatal differentiation programs [45]. A very recent transcriptome analysis of stomatal precursor cells suggests that the stomatal initial cells express trichome regulators and have the potential to give rise to trichome cell fate [46]. It is an exciting future area of research to understand how the production and diffusion of both stomatal and trichome regulators co-exist in a given cell so as to initiate patterning and eventually cause bifurcation of cell fate.

Historically, stomatal development was briefly introduced, together with insect bristle patterning, as an example of two-dimensional periodic pattern generated by hypothetical activators and inhibitors by H. Meinhardt in 1982 [30]. This was based on A. Turing’s model published over 60 years ago [31]. Our work suggests that the network wiring of regulatory components initiating stomatal patterning highlights a simple and conserved logic of pattern formation using a variation of the reaction-diffusion systems also found in the animal development system [47–50]. Understanding how this circuit intersects with cell division, polarity, and growth in the context of whole-leaf development may offer a broader perspective on how genes and regulatory pathways control the overall shape and patterning.

## Materials and Methods

### Plant materials and growth conditions

The *Arabidopsis* ecotype Columbia (Col) was used as wild type. The following mutants and reporter transgenic plant lines were reported previously: *spch-3* and *SPCHpro*::*SPCH-GFP* [2]; *scrm-D*, *scrm scrm2*, *SCRMpro*::*GFP-SCRM*, *SCRMpro*::*GFP-scrm-D* [4]; *tmm-KO*, *epf2-1*, and *EPF2pro*::*erGFP* [7]; *AtML1pro*::*NLS-3xGFP* [51], *er-105 erl1-2 erl2-1* [5]; *TMMpro*::*TMM-YFP*, *ERECTApro*::*ERECTA-ΔK* [9] and *ERECTApro*::*ERECTA-YFP* [9]. Reporter lines were introduced into respective mutant backgrounds by genetic crosses, and genotypes were confirmed by PCR. Seedlings and plants were grown as described previously [9]. PCR-based genotyping of mutants was done using primers listed in S1 Table. Bikinin treatment was done as previously published [22].

### Plasmid construction and transgenic plants generation

The following plasmids were constructed: pAR130 (*SCRM* promoter cassette), pAR132 (nucGFP cassette), pAR152 (*SCRMpro*::*nucGFP*), pAR175 (*SPCH* promoter cassette), pAR200 (*SPCHpro*::*nucGFP*), pJT156 (*SCRM2* promoter cassette), pJT160 (GFP-SCRM2), pJT161 (*SCRM2pro*::*GFP-SCRM2*), pJT167 (*SCRM2prom*::*nucGFP*), pKUT612 (pENTR-D-Keiko), pRJH64 (*EPF2pro*::*LUC*), pLJP246 (estradiol-inducible *SPCH*), and pRJH68 (*SCRMpro*::*LUC*). pCS003 (*TMMpro*::*LUC*), pMK165 (*35S*::*SCRM*) and pLJP152 (*35S*::*SPCH*) were previously published [4,52]. For detailed information about each plasmid, see S2 Table. The primers used for plasmid construction are listed in S1 Table. The nucGFP cassette contains a nuclear localization signal followed by three tandem GFPs. Transgenic Arabidopsis plants were generated by floral dipping. At least five lines per construct were subjected to detailed characterization. Selected reporter lines were crossed with *spch* and *scrm scrm2* mutants.

### Microscopy

Confocal microscopy images were taken using Zeiss LSM700 for GFP as described previously [13]. For receptor-YFP fusions, Leica SP5 was used with White Light Laser (excitation at 518 nm and emission at 540 nm for EYFP; excitation 619 nm and emission at 642 nm for propidium iodide) using HyD detector. Cell peripheries were visualized with either propidium iodide (Molecular Probes) or FM4-64 (Invitrogen). The confocal images were false colored, and brightness/contrast were adjusted using Photoshop CS6 (Adobe). The line scan analysis was performed using ImageJ64.

### Chromatin immunoprecipitation (ChIP)

SPCH-GFP fusion protein does not abundantly accumulate in leaf epidermis. To enrich for stomatal-lineage cells, we introduced *SPCHpro*::*SPCH-GFP* to *scrm-D* mutant, which causes nearly all epidermal cells to adopt stomatal-lineage cell fate [4]. Transcriptomic profiling has confirmed that the genome-wide *scrm-D* effects are highly specific to stomatal differentiation pathways [13]. Likewise, transgenic seedlings expressing wild-type *SCRMpro*::*GFP-SCRM*, *SCRM2pro*::*GFP-SCRM2*, as well as its gain-of-function version *SCRMpro*::*GFP-scrm-D* was used for ChIP assays. *SCRMpro*::*GFP-scrm-D* confers stomata-only epidermis [4]. Five or 12-day-old seedlings were harvested in the middle of the light cycle. Procedures for cross-linking and chromatin isolation were performed as previously described [53]. DNA was sheared by sonication to yield an average fragment size of 200–1000 bp using Bioruptor Plus UCD-300 sonicator (Diagenode). Immunoprecipitation was performed by over night incubation with Dynabeads protein G (Invitrogen) pre-coated with anti-GFP (Abcam A290) antibody at 4°C. Immunocomplexes were washed subsequently in low salt, high salt, LiCl and TE buffer according to Bowler et al., 2004 [53], eluted and revere cross-linked in 10% Chelex (BioRad) at 95°C, and treated with Proteinase K for 30 min at 50°C followed by incubation at 95°C for 10 min. DNA fragments were purified using a PCR purification kit (Qiagen). 1 μL of precipitated DNA were used as templates with primers listed in S3 Table. Input samples were diluted 1:1000 before qPCR analysis. Enrichment of specific amplicons was calculated using the Pfaffl method [54]. For each analysis, at least three biological replicates were performed.

### Real-time polymerase chain reaction (qRT-PCR)

Isolation of RNA and cDNA preparation as done as described previously [13]. PCR was performed using a CFX96 real-time PCR detection system (Bio-Rad) with Power SYBR Green Mastermix (Applied Biosystems). Data was normalized against ACT2 and relative expression calculated using the Pfaffl method [54]. For primer information, see S1 Table.

### Bioassays of recombinant MEPF2 peptide

Expression, purification, and refolding of MEPF2 peptides were performed as described previously [9]. For bioassays, either buffer alone (mock: 50 mM Tris-HCl at pH 8.0) or refolded recombinant MEPF peptides (1 μM) in buffer were applied to 1-day-old Arabidopsis seedlings. After 5 days of further incubation in MS liquid medium containing each peptide, stomatal phenotypes were determined by confocal microscopy.

### Dual luciferase transactivation assay *in planta*

Dual luciferase transactivation assays were done in biological triplicate by *Agrobacterium*-infiltration of 4–5 week old *N*. *benthamiana* leaves as described previously [52]. Five to seven days after infiltration, firefly luciferase (LUC) and *Renilla* luciferase (REN) were assayed using dual luciferase reagents (Promega) and measured using a Victor^3^ V Plate Reader. See S1 Table and S2 Table for details about plasmid construction and oligo DNA sequences used.

### Mathematical modeling

Based on our experimental observations, a series of ordinary differential equations that describe the concentration changes of SPCH, SCRM, SPCH•SCRM heterodimers, EPF2, and TMM were described (see Eqs (3)-(7) in S1 Text). Based on the experimental observations, parameters for ER and Stomagen were set at constant levels. Both ER•ER homodimers and ER•TMM heterodimers have been shown to associate with EPF2 and Stomagen (manuscript currently under review), thus signaling output was simulated by incorporating all possible combination of receptor dimers with/without ligands (see Eqs. (10)-(33) in S1 Text). Numerical simulations are calculated by Euler’s method with a time step *Δt* = 0.002 using Eqs. (2)-(9) and (31)-(33), until total time reaches *t* = 2000.0 where patterns no longer change. Hexagonal cells are two-dimensionally arranged with the periodic boundary condition. Initial values of variables are given as their equilibrium with random fluctuation of 10.0%. For full, formal description of regulatory networks and mathematical definitions of each component, see S1 Text.

## Supporting Information

S1 Text Complete formal description of regulatory networks and mathematical definitions of each model component. Includes a detailed description of the modeling framework with details on individual model components and parameters, as well as additional references cited in this file that are not cited in the main text.
(PDF)

S1 Table List of primers and their DNA sequence used in this study. (XLS)

S2 Table List of Plasmids constructed in this study. (XLS)

S3 Table Primer sequence for ChIP Assays. (XLSX)

S1 Fig Accumulation of functional GFP fusion proteins of SPCH, SCRM, and SCRM2 in the presence or absence of *SPCH* and *SCRMs*. Shown are confocal microscope images of abaxial epidermis from the early protoderm (**A, B, E, F, I, J**) and developing rosette leaves (**C, D, G, H, K, L**) of 10-12-day-old *Arabidopsis* seedlings expressing *SPCHpro*::*SPCH-GFP* (**A-D**), *SCRMpro*::*GFP-SCRM* (**E-H**), and *SCRM2pro*::*GFP-SCRM2* (**I-L**). These functional GFP-fused constructs are in wild-type (**A, C, E, G, I, K**) or the opposite knockout mutant backgrounds (*SPCHpro*::*SPCH-GFP* in *scrm scrm2* [**B, D**]; *SCRMpro*::*GFP-SCRM* and *SCRM2pro*::*GFP-SCRM2* in *spch* [**F, H, J, L**]). Note that introduction of *SPCHpro*::*SPCH-GFP* into *spch*, or *SCRMpro*::*GFP-SCRM* or *SCRM2pro*::*GFP-SCRM2* into *scrm scrm2* rescues the pavement-cell-only mutant phenotypes and therefore cannot be used to investigate the expression of these bHLH proteins in the absence of stomatal-lineage initiation. SPCH-GFP is accumulating in a subset of protodermal cells (**A, B**; dots) as well in meristemoids (**A, C**; asterisks). In some instances, SPCH-GFP is detected in dividing protodermal cells in *scrm scrm2* despite the absence of stomatal cell lineages (**B;** dots). In wild type, GFP-SCRM and GFP-SCRM2 are detected in a subset of protodermal cells (**E, I;** dots), meristemoids (**E, G, I, K;** asterisks) and guard mother cells (**G, K;** arrowheads); GFP-SCRM signal remains strong in immature guard cells (g; pluses) and mature guard cells. No GFP-SCRM or GFP-SCRM2 proteins are detected in *spch* mutant background (**F, H, J, L**). Scale bars, 20 μm.
(TIF)

S2 Fig ChIP data expressed in percent input. Shown are the same ChIP data as in the main figures (Fig 1D and 1E, Fig 2B, and Fig 3D), but presented as % input. For the location of each amplicon, see main figures.
(TIF)

S3 Fig The initial regulatory circuit model simulating two-dimensional patterns of stomatal initial cells. (**A**) Diagram of regulatory circuit used for the initial modeling. (Left) wild type (wt). (Right) *erecta*-triple mutant. Arrow designates activation and T-bar designates inhibition. Double arrowheads between EPF2, ER, and TMM indicate combinatorial ligand-receptor associations (see S7 Fig and S1 Text). Concentrations of each components are abbreviated as the following: *u*_1_, SPCH; *u*_2_, SCRM; *u*_3_, SPCH•SCRM heterodimer; *v*_1_, EPF2; *w*, TMM. *m*, strength of MAPK-mediated inhibition. (Right) Based on this initial model, MAPK cascade will not be activated in the absence of *ERECTA*-family, and this results in the entire epidermis adopting stomatal precursor identity. (**B**) Confocal microscopy of a primary rosette leaf protoderm from one-week-old seedlings of wild-type (left) and *erecta*-triple mutant (right) expressing *SCRMpro*::*GFP-SCRM*. Nuclear accumulation of GFP-SCRM (arrowheads) are spread out in the wild-type protoderm (left), while they are clustered and in *erecta*-triple mutant (right). Images were taken under the same magnification. (**C**) Initial spatial patterns of each component in wild type, *tmm*, and *erecta*-family triple mutant simulated *in silico* based on the mathematical models. Each square represents a sheet of protoderm with 400 cells (each cell represented by a hexagon). White cells indicate no expression/accumulation of a given component, while dark blue cells express/accumulate high amounts. In *erecta*-triple mutant, all epidermal cells become stomatal initials, which is not consistent with the observed phenotype. (**D**) Sensitivity of wild-type and *tmm* protoderm to EPF2 application *in silico*. *C*_0_ designates the concentration of exogenously applied EPF2 (MEPF2) peptide. In this model, *tmm* is less sensitive to EPF2 than wild type, but the stomatal differentiation can still be inhibited by exogenously applied MEPF2 peptide, which is not consistent with the observed phenotype (see S4 Fig).
(TIF)

S4 Fig Epidermal phenotypes of stomatal mutants and transgenic plants. Confocal images of 1-to-2-week-old abaxial cotyledon epidermis. (**A**) wild type (wt) and other stomatal mutants simulated in this study (*spch*, *scrm-D*, *scrm scrm2*, *er erl1 erl2 and tmm*). (**B**) Phenotypic similarity among the activator and inhibitor of stomatal initiation. Shown are confocal images of 5-day-old cotyledon abaxial epidermis. Loss of EPF2 or ERECTA signaling by introduction of a dominant-negative form of ERECTA (ER∆Kinase in *erecta*) confers a phenotype similar to that of ectopic *SPCH* overexpression (*SPCH-OX*). All these plants show excessive entry into stomatal cell lineages (yellow brackets). Scale bar, 20 μm.
(TIF)

S5 Fig Including signaling ligand outside of the SPCH•SCRM module correctly predicts the phenotypic consequence of EPF2 peptide application. (**A**) Spatial patterns of stomatal initial cells in wild type and *tmm* mutants, with increasing amounts of exogenous EPF2 peptide (*C*_0_) simulated *in silico*. Each square represents a sheet of protoderm with 400 cells (each cell represented by a hexagon). The stomatal initial cells are marked by SPCH•SCRM heterodimers (*u*_3_), with no expression shown as white and maximal expression as dark blue. (**B**) Application of predicted, mature EPF2 (MEPF2) peptide show no effects on *tmm* stomatal cluster phenotype. Images of cotyledons from 6-day-old seedlings were taken under the same magnification. Scale bar, 40 μm. (**C**) Initial model explaining the sensitivity of *tmm* to EPF2 application. Increased EPF2 triggers inhibitory signals through ERECTA, which activates downstream MAPK cascade and inhibits stomatal differentiation. (**D**) Revised model explaining insensitivity of *tmm* to EPF2 application. Here, the presence of a signal (likely Stomagen) that competes with EPF2, balances the activity of ERECTA and maintains the signaling output in the absence of TMM.
(TIF)

S6 Fig Epidermal phenotypes of *scrm-D* and *erecta-*triple mutant seedlings in response to bikinin treatment. *scrm-D* and *erecta-*triple mutant (*er erl1 erl2*) seedlings were germinated and grown for 8 days on 30 μM bikinin (right panels) or without bikinin (left panels). (**A**) Representative confocal microscopy images of cotyledon abaxial epidermis. As reported previously [22], bikinin treatment confers no effects on *scrm-D* stomata-only phenotypes, while stomatal clustering phenotype of *er erl1 erl2* gets alleviated. (**B**) Representative confocal microscopy images of hypocotyl epidermis. Bikinin treatment reduces stomatal clusters in *scrm-D* and, surprisingly, completely suppress stomatal differentiation in *er erl1 erl2*. Scale bar, 20 μm.
(TIF)

S7 Fig Effects of *tmm* and *erecta*-family triple null mutations on the regulatory circuit simulated in this study. (**A-C**) Top: Shown are regulatory circuit diagrams of wild type (WT: **A**), *tmm* (**B**), and *erecta* (*er*)-family triple (**C**) mutant used for our mathematical modeling presented in Fig 4 and S4 Fig. Concentrations of each component are abbreviated as the following: *u*_1_, SPCH; *u*_2_, SCRM; *u*_3_, SPCH•SCRM heterodimer; *v*_1_, EPF2; *w*, TMM; *v*_2_, EPF2-independent hypothetical component. *m*, strength of MAPK-mediated inhibition. *S*, a component that competes for receptor pools, most likely Stomagen. Bottom: Available ligand-receptor pools and additional components that activate MAPK to inhibit stomatal initials in each scenario. (**D**) Example of numerical solution of the ligand-receptor complex concentrations for Eqs. (19)–(28) in S1 Text with parameter condition of *k*_1_ = *k*_2_ = 1.0, *k*_3_ = *k*_4_ = *k*_5_ = *k*_6_ = 2.0, *E*_0_ = 3.5, *S*_0_ = 3.0, and *w* = 1.0. (**E**) Increasing and decreasing changes in concentrations of ligand-receptor complexes (D) are approximated by *x*/(*K* + *x*) and *K*/(*K* + *x*), respectively, in our model.
(TIF)

S8 Fig Effects of diffusion constants of the inhibitors and activators on stomatal lineage patterns *in silico*. Shown are simulations of stomatal lineage initiation patterns calculated with a range (2.0–100.0) of the ratio of diffusion constants (*d* = *d*_*v*_/*d*_*u*_) of inhibitors (*d*_*v*_ = *d*_v1_ = *d*_v2_) over activators (*d*_*u*_ = *d*_u1_ = *d*_u2_ = 0.02) in a function of a range (0.05–5.0) of parameter *G*. *G* is a reaction rate coefficient of the negative feedback loop (see Eqs. (6)–(8) in S1 Text). As a value of *G* decreases, the optimal value for *d* for proper stomatal patterning decreases. Highlighted in pink rectangle is our standard simulation condition of *G* = 1.0 and *d* = 100.0 (see S1 Text). Blue, cells accumulating SPCH•SCRMs (*u*_3_); White, cells with no expression/accumulation.
(TIF)

S9 Fig Effects of cooperativity of SPCH/SCRM degradation and SPCH•SCRM-mediated gene expression on stomatal lineage patterns *in silico*. Shown are simulations of stomatal lineage initiation patterns when Hill coefficients for parameters *p* (cooperativity of *SCRMs*, *EPF2* and *TMM* gene expression by SPCH•SCRMs) and *q* (cooperativity of SPCH and SCRM protein degradation) are altered. Here simulations were done with *p* = 1.0–3.0 and *q* = 1.0–5.0. See Eqs. (3)–(8) for parameters *p* and *q* (S1 Text). Values for cooperativity *p* ranging between 1.4 and 2.6 (when *q* = 3.0) are required for spatial patterning of stomatal initials. Conditions that create any stomatal-lineage initials are highlighted in blue.
(TIF)

S10 Fig Effects of *tmm* mutation on cotyledon epidermal phenotypes of *erecta*-family higher order mutants. Shown are representative confocal microscopy images of abaxial cotyledon epidermis from seven-day-old seedlings of: (**A**) wild type (wt), (**B**) *tmm*, (**C**) *er*, (**D**) *tmm er*, (**E**) *erl1*, (**F**) *tmm erl1*, (**G**) *erl2*, (**H**) *tmm erl2*, (**I**) *er erl1*, (**J**) *tmm er erl1*, (**K**) *er erl2*; (**L**) *tmm er erl2*; (**M**) *erl1 erl2*; (**N**) *tmm erl1 erl2*; (**O**) *er erl1 erl2*; (**P**) *tmm er erl1 erl2*. The cotyledons from any combination of *er-*family higher order mutants with additional *tmm* mutation exhibit stomatal clusters. Scale bars, 50 μm.
(TIF)

S11 Fig Effects of *tmm* mutation on protodermal phenotypes of *erecta*-family higher order mutants. Shown are representative confocal microscopy images of abaxial rosette leaf protoderm from 7 or 8-day-old seedlings of: (**A**) wild type (wt), (**B**) *tmm*, (**C**) *er*, (**D**) *tmm er*, (**E**) *erl1*, (**F**) *tmm erl1*, (**G**) *erl2*, (**H**) *tmm erl2*, (**I**) *er erl1*, (**J**) *tmm er erl1*, (**K**) *er erl2*; (**L**) *tmm er erl2*; (**M**) *erl1 erl2*; (**N**) *tmm erl1 erl2*; (**O**) *er erl1 erl2*; (**P**) *tmm er erl1 erl2*. The additional *tmm* mutation appears to increase meristemoids in *er-*family higher order mutants. Scale bars, 50 μm.
(TIF)

S12 Fig Effects of a synthesis rate of TMM and synthesis rate of SPCH on stomatal lineage patterns *in silico*. Shown are simulations of stomatal lineage initiation patterns in the range of a synthesis rate of TMM (*C*_3_, 0.0–10.0) and that of SPCH (*A*_1_, 0.2–20.0). See Eqs. (7) and (3) for *C*_3_ and *A*_1_, respectively (S1 Text). Synthesis rate of SPCH greatly influences distribution and patterning of stomatal initial cells, whereas synthesis rate of TMM has a modest role in enforcing spacing. Blue, high levels of SPCH•SCRMs (*u*_3_); White, cells with no expression/accumulation. Highlighted in pink rectangle is our standard simulation condition of *A*_1_ = 2.0 and *C*_3_ = 1.0.
(TIF)

S13 Fig Introduction of noise has little influence on stomatal lineage patterns *in silico*. Shown are five independent simulations with random noise being introduced to the initial values of all components (percentages of noise are indicated above each column). Shown in the top row are representative of SPCH•SCRMs (*u*_*3*_) initial distribution in two-dimensional space (20 x 20 hexagons per each condition) upon introduction of respective noise. Shown in blue are cells expressing SPCH•SCRMs; white—no expression.
(TIF)

## References

[1] MacAlister CA, Ohashi-Ito K, Bergmann DC (2007) Transcription factor control of asymmetric cell divisions that establish the stomatal lineage. Nature 445: 537–540. 17183265 10.1038/nature05491

[2] Pillitteri LJ, Sloan DB, Bogenschutz NL, Torii KU (2007) Termination of asymmetric cell division and differentiation of stomata. Nature 445: 501–505. 17183267 10.1038/nature05467

[3] Ohashi-Ito K, Bergmann DC (2006) Arabidopsis FAMA controls the final proliferation/differentiation switch during stomatal development. Plant Cell 18: 2493–2505. 17088607 10.1105/tpc.106.046136 PMC1626605

[4] Kanaoka MM, Pillitteri LJ, Fujii H, Yoshida Y, Bogenschutz NL, et al (2008) SCREAM/ICE1 and SCREAM2 specify three cell-state transitional steps leading to arabidopsis stomatal differentiation. Plant Cell 20: 1775–1785. 10.1105/tpc.108.060848 18641265 PMC2518248

[5] Shpak ED, McAbee JM, Pillitteri LJ, Torii KU (2005) Stomatal patterning and differentiation by synergistic interactions of receptor kinases. Science 309: 290–293. 16002616 10.1126/science.1109710

[6] Hara K, Kajita R, Torii KU, Bergmann DC, Kakimoto T (2007) The secretory peptide gene EPF1 enforces the stomatal one-cell-spacing rule. Genes Dev 21: 1720–1725. 17639078 10.1101/gad.1550707 PMC1920166

[7] Hara K, Yokoo T, Kajita R, Onishi T, Yahata S, et al (2009) Epidermal cell density is auto-regulated via a secretory peptide, EPIDERMAL PATTERNING FACTOR2 in Arabidopsis leaves. Plant Cell Physiol 50: 1019–1031. 10.1093/pcp/pcp068 19435754

[8] Hunt L, Gray JE (2009) The signaling peptide EPF2 controls asymmetric cell divisions during stomatal development. Curr Biol 19: 864–869. 10.1016/j.cub.2009.03.069 19398336

[9] Lee JS, Kuroha T, Hnilova M, Khatayevich D, Kanaoka MM, et al (2012) Direct interaction of ligand-receptor pairs specifying stomatal patterning. Genes Dev 26: 126–136. 10.1101/gad.179895.111 22241782 PMC3273837

[10] Bergmann DC, Lukowitz W, Somerville CR (2004) Stomatal development and pattern controlled by a MAPKK kinase. Science 304: 1494–1497. 15178800 10.1126/science.1096014

[11] Wang H, Ngwenyama N, Liu Y, Walker J, Zhang S (2007) Stomatal development and patterning are regulated by environmentally responsive mitogen-activated protein kinases in Arabidopsis. Plant Cell 19: 63–73. 17259259 10.1105/tpc.106.048298 PMC1820971

[12] Lampard GR, Macalister CA, Bergmann DC (2008) Arabidopsis stomatal initiation is controlled by MAPK-mediated regulation of the bHLH SPEECHLESS. Science 322: 1113–1116. 10.1126/science.1162263 19008449

[13] Pillitteri LJ, Peterson KM, Horst RJ, Torii KU (2011) Molecular profiling of stomatal meristemoids reveals new component of asymmetric cell division and commonalities among stem cell populations in Arabidopsis. Plant Cell 23: 3260–3275. 10.1105/tpc.111.088583 21963668 PMC3203429

[14] Robinson S, Barbier de Reuille P, Chan J, Bergmann D, Prusinkiewicz P, et al (2011) Generation of spatial patterns through cell polarity switching. Science 333: 1436–1440. 10.1126/science.1202185 21903812 PMC3383840

[15] Peterson KM, Rychel AL, Torii KU (2010) Out of the mouths of plants: the molecular basis of the evolution and diversity of stomatal development. Plant Cell 22: 296–306. 10.1105/tpc.109.072777 20179138 PMC2845417

[16] Rowe MH, Bergmann DC (2010) Complex signals for simple cells: the expanding ranks of signals and receptors guiding stomatal development. Curr Opin Plant Biol 13: 548–555. 10.1016/j.pbi.2010.06.002 20638894 PMC2967594

[17] Rychel AL, Peterson KM, Torii KU (2010) Plant twitter: ligands under 140 amino acids enforcing stomatal patterning. J Plant Res 123: 275–280. 10.1007/s10265-010-0330-9 20336477

[18] Nadeau JA, Sack FD (2002) Control of stomatal distribution on the Arabidopsis leaf surface. Science 296: 1697–1700. 12040198 10.1126/science.1069596

[19] Torii KU (2012) Two-dimensional spatial patterning in developmental systems. Trends Cell Biol 22: 438–446. 10.1016/j.tcb.2012.06.002 22789547

[20] Kondo S, Miura T (2010) Reaction-diffusion model as a framework for understanding biological pattern formation. Science 329: 1616–1620. 10.1126/science.1179047 20929839

[21] Gierer A, Meinhardt H (1972) A theory of biological patten formation. Kybernetik 12: 30–39. 4663624 10.1007/BF00289234

[22] Kim TW, Michniewicz M, Bergmann DC, Wang ZY (2012) Brassinosteroid regulates stomatal development by GSK3-mediated inhibition of a MAPK pathway. Nature 482: 419–422. 10.1038/nature10794 22307275 PMC3292258

[23] Gudesblat GE, Schneider-Pizon J, Betti C, Mayerhofer J, Vanhoutte I, et al (2012) SPEECHLESS integrates brassinosteroid and stomata signalling pathways. Nat Cell Biol 14: 548–554. 10.1038/ncb2471 22466366

[24] Khan M, Rozhon W, Bigeard J, Pflieger D, Husar S, et al (2013) Brassinosteroid-regulated GSK3/Shaggy-like kinases phosphorylate mitogen-activated protein (MAP) kinase kinases, which control stomata development in Arabidopsis thaliana. J Biol Chem 288: 7519–7527. 10.1074/jbc.M112.384453 23341468 PMC3597792

[25] De Rybel B, Audenaert D, Vert G, Rozhon W, Mayerhofer J, et al (2009) Chemical inhibition of a subset of Arabidopsis thaliana GSK3-like kinases activates brassinosteroid signaling. Chem Biol 16: 594–604. 10.1016/j.chembiol.2009.04.008 19549598 PMC4854203

[26] Lu P, Porat R, Nadeau JA, O'Neill SD (1996) Identification of a meristem L1 layer-specific gene in Arabidopsis that is expressed during embryonic pattern formation and defines a new class of homeobox genes. Plant Cell 8: 2155–2168. 8989876 10.1105/tpc.8.12.2155 PMC161342

[27] Sugano SS, Shimada T, Imai Y, Okawa K, Tamai A, et al (2010) Stomagen positively regulates stomatal density in Arabidopsis. Nature 463: 241–244. 10.1038/nature08682 20010603

[28] Kondo T, Kajita R, Miyazaki A, Hokoyama M, Nakamura-Miura T, et al (2010) Stomatal density is controlled by a mesophyll-derived signaling molecule. Plant Cell Physiol 51: 1–8. 10.1093/pcp/pcp180 20007289

[29] Ohki S, Takeuchi M, Mori M (2011) The NMR structure of stomagen reveals the basis of stomatal density regulation by plant peptide hormones. Nat Commun 2: 512 10.1038/ncomms1520 22027592

[30] Meinhardt H (1982) Models of biological pattern formation London, UK: Academic Press.

[31] Turing AM (1952) The chemical basis of morphogenesis. Philos Trans R Soc London B 237: 37–72. 10.1098/rstb.2014.0218 PMC4360114 25750229

[32] Lee JS, Hnilova M, Maes M, Lin YCL, Putarjunan A, et al (2015) Competitive binding of antagonistic peptides fine-tunes stomatal patterning. Nature 522:439–43 10.1038/nature14561 26083750 PMC4532310

[33] Abrash EB, Davies KA, Bergmann DC (2011) Generation of Signaling Specificity in Arabidopsis by Spatially Restricted Buffering of Ligand-Receptor Interactions. Plant Cell 23: 2864–2879. 10.1105/tpc.111.086637 21862708 PMC3180797

[34] Torii KU (2012) Mix-and-match: ligand-receptor pairs in stomatal development and beyond. Trends Plant Sci 17: 711–719. 10.1016/j.tplants.2012.06.013 22819466

[35] Uchida N, Lee JS, Horst RJ, Lai H-H, Kajita R, et al (2012) Regulation of inflorescence architecture by intertissue layer ligand-receptor communication between Proc Natl Acad Sci U S A 109: 6337–6342. 10.1073/pnas.1117537109 22474391 PMC3341066

[36] Lau OS, Davies KA, Chang J, Adrian J, Rowe MH, et al (2014) Direct roles of SPEECHLESS in the specification of stomatal self-renewing cells. Science 345: 1605–1609. 10.1126/science.1256888 25190717 PMC4390554

[37] Ayer DE, Kretzner L, Eisenman RN (1993) Mad: a heterodimeric partner for Max that antagonizes Myc transcriptional activity. Cell 72: 211–222. 8425218 10.1016/0092-8674(93)90661-9

[38] Sloan SR, Shen CP, McCarrick-Walmsley R, Kadesch T (1996) Phosphorylation of E47 as a potential determinant of B-cell-specific activity. Mol Cell Biol 16: 6900–6908. 8943345 10.1128/mcb.16.12.6900 PMC231693

[39] Kim TW, Wang ZY (2010) Brassinosteroid signal transduction from receptor kinases to transcription factors. Annu Rev Plant Biol 61: 681–704. 10.1146/annurev.arplant.043008.092057 20192752

[40] Engineer CB, Ghassemian M, Anderson JC, Peck SC, Hu H, et al (2014) Carbonic anhydrases, EPF2 and a novel protease mediate CO control of stomatal development. Nature 513: 246–250. 10.1038/nature13452 25043023 PMC4274335

[41] Hulskamp M (2004) Plant trichomes: a model for cell differentiation. Nat Rev Mol Cell Biol 5: 471–480. 15173826 10.1038/nrm1404

[42] Serna L, Martin C (2006) Trichomes: different regulatory networks lead to convergent structures. Trends Plant Sci 11: 274–280. 16697247 10.1016/j.tplants.2006.04.008

[43] Schnittger A, Folkers U, Schwab B, Jurgens G, Hulskamp M (1999) Generation of a spacing pattern: the role of triptychon in trichome patterning in Arabidopsis. Plant Cell 11: 1105–1116. 10368181 10.1105/tpc.11.6.1105 PMC144244

[44] Bouyer D, Geier F, Kragler F, Schnittger A, Pesch M, et al (2008) Two-dimensional patterning by a trapping/depletion mechanism: the role of TTG1 and GL3 in Arabidopsis trichome formation. PLoS Biol 6: e141 10.1371/journal.pbio.0060141 18547143 PMC2422854

[45] Yan L, Cheng X, Jia R, Qin Q, Guan L, et al (2014) New phenotypic characteristics of three *tmm* alleles in Arabidopsis thaliana. Plant Cell Rep 33: 719–731. 10.1007/s00299-014-1571-1 24553751

[46] Adrian J, Chang J, Ballenger CE, Bargmann BO, Alassimone J, et al (2015) Transcriptome dynamics of the stomatal lineage: birth, amplification, and termination of a self-renewing population. Dev Cell 33: 107–118. 10.1016/j.devcel.2015.01.025 25850675 PMC4390738

[47] Kondo S, Asai R (1995) A reaction-diffusion wave on the skin of the marine angelfish Pomacanthus. Nature 376: 765–768. 24547605 10.1038/376765a0

[48] Inaba M, Yamanaka H, Kondo S (2012) Pigment pattern formation by contact-dependent depolarization. Science 335: 677 10.1126/science.1212821 22323812

[49] Muller P, Rogers KW, Jordan BM, Lee JS, Robson D, et al (2012) Differential diffusivity of Nodal and Lefty underlies a reaction-diffusion patterning system. Science 336: 721–724. 10.1126/science.1221920 22499809 PMC3525670

[50] Sheth R, Marcon L, Bastida MF, Junco M, Quintana L, et al (2012) Hox genes regulate digit patterning by controlling the wavelength of a Turing-type mechanism. Science 338: 1476–1480. 10.1126/science.1226804 23239739 PMC4486416

[51] Takada S, Jurgens G (2007) Transcriptional regulation of epidermal cell fate in the Arabidopsis embryo. Development 134: 1141–1150. 17301085 10.1242/dev.02803

[52] Peterson KM, Shyu C, Burr CA, Horst RJ, Kanaoka MM, et al (2013) Arabidopsis homeodomain-leucine zipper IV proteins promote stomatal development and ectopically induce stomata beyond the epidermis. Development 140: 1924–1935. 10.1242/dev.090209 23515473 PMC3631968

[53] Bowler C, Benvenuto G, Laflamme P, Molino D, Probst AV, et al (2004) Chromatin techniques for plant cells. Plant J 39: 776–789. 15315638 10.1111/j.1365-313X.2004.02169.x

[54] Pfaffl MW (2001) A new mathematical model for relative quantification in real-time RT-PCR. Nucleic Acids Res 29: e45 11328886 10.1093/nar/29.9.e45 PMC55695

