# Supplementary material for: Molecular Framework of a Regulatory Circuit Initiating Two-Dimensional Spatial Patterning of Stomatal Lineage
Source: PLoS Genet. 2015 Jul 23;11(7):e1005374. doi: 10.1371/journal.pgen.1005374 (PMC4512730; doi:10.1371/journal.pgen.1005374)
Supplement: S1 Text — Includes a detailed description of the modeling framework with details on individual model components and parameters, as well as additional references cited in this file that are not cited in the main text. (PDF) [file pgen.1005374.s001.pdf]

S1 Text

Supplementary Methods

Model framework

I. Overview

This supplementary method provides a basic framework for modeling regulatory circuits initiating stomatal precursor cells. Here, each protodermal cell is represented by the hexagons, and each cell has an equal potential to differentiate into a stomatal initial cell. Based on the experimental observations reported in this manuscript and those by others (REF), we make a series of assumptions: (a) SPCH and SCRM form heterodimers; (b) SPCH is constitutively expressed and degraded by MAPK signaling; (c) SCRM is produced by SPCH•SCRM heterodimers and degraded by MAPK signaling; (d) *EPF2* expression is regulated by SPCH•SCRM; (e) EPF2 diffuse at substantially faster rate than SPCH•SCRM; (e) TMM expression is regulated by SPCH•SCRM; (e) MAPK cascade is activated by ER and TMM, and ER forms homodimers or heterodimers with TMM; (f) this MAPK cascade leads to SPCH•SCRM degradation; and (g) an EPF2-independent MAPK cascade also leads to SPCH•SCRM degradation. See Fig 4 for the diagrams of regulatory circuits and simulated outcomes. The following are step-by-step descriptions of our modeling assumptions and actual mathematical equations used.

II. Regulatory relationships among SPCH, SCRM, EPF2, and TMM

We make the following assumptions based on experimental observations to describe the concentration changes of molecules in the *i*-th cell.

(i) SPCH (u_1) and SCRM (u_2) associate to form the heterodimer SPCH•SCRM (u_3) according to a reversible reaction:

$$\mathrm{SPCH} + \mathrm{SCRM} \rightleftarrows \mathrm{SPCH} \bullet \mathrm{SCRM}. \tag{1}$$

In this reaction, the increasing rate of u_3 concentration (equivalent to the decreasing rate of u_1 or u_2 concentration) is described by

$$\Delta = k_+ u_1 u_2 - k_- u_3, \tag{2}$$

where k_+ and k_- are the association and dissociation rate constants, respectively.

(ii) SPCH (u_1) is constitutively synthesized, reacts according to reaction (1), is degraded depending on MAPK signaling (m), and diffuses between cell i and neighboring cell j:

$$\frac{du_{1i}}{dt} = A_1 - \Delta - \left(B_1 + B_{1m} \frac{m^q}{K_m^q + m^q} \right) u_{1i} + d_{u1} \sum_j (u_{1j} - u_{1i}), \tag{3}$$

where A_1 is the synthesis rate, B_1 is the background decay rate, B_{1m} is the coefficient of MAPK-dependent degradation, K_m is the half-saturation coefficient of m, and d_{u1} is the coupling rate between neighboring cells.

(iii) SCRM (u_2) is synthesized in response to SPCH•SCRM (u_3), reacts according to reaction (1), is degraded depending on the MAPK signaling (m), and diffuses between cell i and neighboring cell j:

$$\frac{du_{2i}}{dt} = A_0 + A_2 \frac{u_{3i}^p}{K_0^p + u_{3i}^p} - \Delta - \left(B_2 + B_{2m} \frac{m^q}{K_m^q + m^q} \right) u_{2i} + d_{u2} \sum_j (u_{2j} - u_{2i}), \tag{4}$$

where A_0 is the background synthesis rate, A_2 is the synthesis coefficient, K_0 is the half-saturation coefficient of u_3, B_2 is the background decay rate, B_{2m} is the coefficient of MAPK-dependent degradation, K_m is the half-saturation coefficient of m, and d_{u2} is the coupling rate between neighboring cells.

In comparison to secreted peptide, EPF2, the nuclear localized SPCH and SCRM are likely have limited diffusion. We introduced 1/100 of the coupling rates to u_1 and u_2. It has been shown experimentally that enhanced diffusion in SPCH affect stomatal patterning[55], while the underlying mechanism is beyond the scope of this study.

(iv) SPCH•SCRM (u_3) reacts according to reaction (1) and is degraded at a constant rate:

$$\frac{du_{3i}}{dt} = \Delta - B_3 u_{3i}, \quad (5)$$

where B_3 is the decay rate.

(v) EPF2 (v_1) is synthesized in response to SPCH•SCRM (u_3), is degraded at a constant rate, and diffuses between cell i and neighboring cell j:

$$\frac{dv_{1i}}{dt} = C_0 + G\left[C_1 \frac{u_{3i}^p}{K_1^p + u_{3i}^p} - D_1 v_{1i}\right] + d_{v1} \sum_j (v_{1j} - v_{1i}), \quad (6)$$

where G is the reaction rate coefficient of negative feedback loop, C_1 is the synthesis coefficient, K_1 is the half-saturation coefficient of u_3, D_1 is the decay rate, and d_{v1} is the coupling rate between neighboring cells. C_0 indicates the concentration of exogenously applied EPF2 peptide (S3 Fig D and S5 Fig A). For simplicity our model does not include peptide-processing steps, such CO_2-induced upregulation of EPF2 peptide cleavage reported very recently [37], as a function of activity.

(vi) TMM (w) is synthesized in response to SPCH•SCRM (u_3) and is degraded at a constant rate:

$$\frac{dw_i}{dt} = G\left[C_3 \frac{u_{3i}^p}{K_3^p + u_{3i}^p} - D_3 w_i\right], \quad (7)$$

where C_3 is the synthesis coefficient, K_3 is the half-saturation coefficient of u_3, and D_3 is the decay rate.

We found that EPF2 peptide application triggered loss of SPCH and SCRM protein accumulation and hence the epidermis lacking stomatal-lineage cells (Fig 2D). Together with previous reports [11,12,56], the following can be proposed. The regulatory framework described in the above includes two feedback loops: a positive one by SPCH/SCRM and negative one by EPF2, which are expected to play central roles in patterning of stomatal differentiation. In fact, this regulatory relationship is equivalent to that of the activator–inhibitor system, one of well-known theoretical

models for pattern formation, which can generate stable patterns from a homogeneous state. In this framework, SPCH/SCRM and EPF2 correspond to the activator and inhibitor, respectively.

Thus, if this negative feedback regulation involving EPF2 is completely eliminated, every epidermal cell will highly express SPCH/SCRM and be differentiated into the stomatal lineage. This is the case for *scrm-D* mutation (Figs 2 and 4)[4]. However, despite the complete lack of this signaling pathway, *er erl1 erl2* triple mutant shows clustered stomata, a phenotype weaker than that of *scrm-D*. This strongly indicates the presence of another negative feedback loop not involving EPF2. We accordingly introduce another diffusible molecule with a function similar to EPF2 (v_1).

(vii) An assumed molecule (v_2) is synthesized in response to SPCH•SCRM (u_3), is degraded at a constant rate, and diffuses between cell *i* and neighboring cell *j*:

$$\frac{dv_{2i}}{dt} = G\left[C_2 \frac{u_{3i}^p}{K_2^p + u_{3i}^p} - D_2 v_{2i}\right] + d_{v2}\sum_j (v_{2j} - v_{2i}), \qquad (8)$$

where C_2 is the synthesis coefficient, K_2 is the half-saturation coefficient of u_3, D_2 is the decay rate, and d_{v2} is the coupling rate between neighboring cells.

(viii) Expression level of reporter *SPCHpro::GFP* (g_u) is activated in the same manner as SPCH (u_1, Eq. (3)) and is degraded at a constant rate:

$$\frac{dg_{ui}}{dt} = A_1 - B_g g_{ui}, \qquad (9)$$

where B_g is the decay rate.

(ix) Expression level of reporter *EPF2pro::GFP* (g_v) is activated in the same manner as EPF2 (v_1, Eq. (6)) and is degraded at a constant rate:

$$\frac{dg_{vi}}{dt} = C_1 \frac{u_{3i}^p}{K_1^p + u_{3i}^p} - D_g g_{vi}, \qquad (10)$$

where D_g is the decay rate.

III. Regulation of MAPK signaling activity

We now consider the MAPK signaling activity (m) that is induced by two pathways, EPF2 (v_1)-dependent and v_2-dependent:

$$m = m_1 + v_2 \,, \tag{11}$$

where m_1 indicates the effect of the EPF2-dependent pathway, which involves two ligands (EPF2 and S, which is most likely Stomagen) and two receptor dimers constituted by ERECTA (ER) receptor kinase and TOO MANY MOUTHS (TMM) receptor-like protein (ER•ER homodimer and ER•TMM heterodimer). ER has been shown to both homodimerize with itself and heterodimerize with TMM[9]. In contrast, TMM does not form homodimers[9]. Thus, four ligand-receptor combinations exist: ER•ER•EPF2, ER•ER•Stomagen, ER•TMM•EPF2, and ER•TMM•Stomagen. Therefore, m_1 corresponds to the sum of effects of these ligand-receptor complexes:

$$\begin{aligned} m_1(v_1, w) = {} & c_1[\text{ER•ER•EPF2}] + c_2[\text{ER•ER•Stomagen}] \\ & + c_3[\text{ER•TMM•EPF2}] + c_4[\text{ER•TMM•Stomagen}] \end{aligned} \tag{12}$$

where c_1, c_2, c_3, and c_4 are constants that correspond to the signaling efficiency of each ligand-receptor complex. Because expressions of ERECTA and Stomagen are not directly influenced by the SPCH•SCRM-EPF2 module (Fig 3) we describe here that ER and Stomagen are expressed at a constant level, m_1 depends on EPF2 (v_1) and TMM (w). Note that here we do not discriminate ER from ERL1 and ERL2, because a mathematical description of their unequal redundancy complicates the simulation without benefit to our effort of identifying minimal regulatory circuits.

In order to determine the concentrations of the ligand-receptor complexes of Eq. (12), we consider the process of each reaction. ER molecules associate with each other to form the homodimer ER•ER, and also with TMM to the heterodimer ER•TMM:

$$\text{ER} + \text{ER} \rightleftarrows \text{ER•ER} \tag{13}$$

$$\text{ER} + \text{TMM} \rightleftarrows \text{ER•TMM} \tag{14}$$

These receptor dimers associate with their ligand EPF2 or Stomagen to form ligand-receptor complexes:

$$\mathrm{ER{\bullet}ER} + \mathrm{EPF2} \rightleftarrows \mathrm{ER{\bullet}ER{\bullet}EPF2} \quad (15)$$

$$\mathrm{ER{\bullet}ER} + \mathrm{Stomagen} \rightleftarrows \mathrm{ER{\bullet}ER{\bullet}Stomagen} \quad (16)$$

$$\mathrm{ER{\bullet}TMM} + \mathrm{EPF2} \rightleftarrows \mathrm{ER{\bullet}TMM{\bullet}EPF2} \quad (17)$$

$$\mathrm{ER{\bullet}TMM} + \mathrm{Stomagen} \rightleftarrows \mathrm{ER{\bullet}TMM{\bullet}Stomagen} \quad (18)$$

If these reactions (13)–(18) are in equilibrium states, then we have

$$[\mathrm{ER{\bullet}ER}] = k_1[\mathrm{ER}]^2 \quad (19)$$

$$[\mathrm{ER{\bullet}TMM}] = k_2[\mathrm{ER}][\mathrm{TMM}] \quad (20)$$

$$[\mathrm{ER{\bullet}ER{\bullet}EPF2}] = k_3[\mathrm{ER{\bullet}ER}][\mathrm{EPF2}] \quad (21)$$

$$[\mathrm{ER{\bullet}ER{\bullet}Stomagen}] = k_4[\mathrm{ER{\bullet}ER}][\mathrm{Stomagen}] \quad (22)$$

$$[\mathrm{ER{\bullet}TMM{\bullet}EPF2}] = k_5[\mathrm{ER{\bullet}TMM}][\mathrm{EPF2}] \quad (23)$$

$$[\mathrm{ER{\bullet}TMM{\bullet}Stomagen}] = k_6[\mathrm{ER{\bullet}TMM}][\mathrm{Stomagen}] \quad (24)$$

where k_1, k_2, k_3, k_4, k_5, and k_6 are equilibrium constants.

ER and Stomagen are constitutively expressed at a constant level. This conservation requirement can be written as

$$\begin{aligned} E_0 = {} & [\mathrm{ER}] + 2[\mathrm{ER{\bullet}ER}] + 2[\mathrm{ER{\bullet}ER{\bullet}EPF2}] + 2[\mathrm{ER{\bullet}ER{\bullet}Stomagen}] \\ & + [\mathrm{ER{\bullet}TMM}] + [\mathrm{ER{\bullet}TMM{\bullet}EPF2}] + [\mathrm{ER{\bullet}TMM{\bullet}Stomagen}] \end{aligned}, \quad (25)$$

$$S_0 = [\mathrm{Stomagen}] + [\mathrm{ER{\bullet}ER{\bullet}Stomagen}] + [\mathrm{ER{\bullet}TMM{\bullet}Stomagen}], \quad (26)$$

where E_0 and S_0 are constants of the total concentration of ER and Stomagen molecules, respectively. In addition, v_1 and w correspond to the total concentrations of EPF2 and TMM molecules, respectively:

$$v_1 = [\mathrm{EPF2}] + [\mathrm{ER{\bullet}ER{\bullet}EPF2}] + [\mathrm{ER{\bullet}TMM{\bullet}EPF2}] \quad (27)$$

$$w = [\mathrm{TMM}] + [\mathrm{ER{\bullet}TMM}] + [\mathrm{ER{\bullet}TMM{\bullet}EPF2}] + [\mathrm{ER{\bullet}TMM{\bullet}Stomagen}]. \quad (28)$$

Thereby, concentrations of ligand-receptor complexes can be calculated by numerically solving Eqs. (19)–(28) (S7 Fig D). As EPF2 (v_1) increases, [ER•ER•EPF2] and [ER•TMM•EPF2] increase and [ER•ER•Stomagen] and [ER•TMM•Stomagen] decrease. On the other hand, [ER•TMM•EPF2] and [ER•TMM•Stomagen] increase by increasing TMM (w), but under such a condition

[ER•ER•EPF2] and [ER•ER•Stomagen] decrease. According to these numerical results, we use simplified forms for these concentrations in our model:

$$[\text{ER•ER•EPF2}] \propto \frac{K_w}{K_w + w}\frac{v_1}{K_v + v_1} \tag{29}$$

$$[\text{ER•ER•Stomagen}] \propto \frac{K_w}{K_w + w}\frac{K_v}{K_v + v_1} \tag{30}$$

$$[\text{ER•TMM•EPF2}] \propto \frac{w}{K_w + w}\frac{v_1}{K_v + v_1} \tag{31}$$

$$[\text{ER•TMM•Stomagen}] \propto \frac{w}{K_w + w}\frac{K_v}{K_v + v_1}, \tag{32}$$

where K_w and K_v are the half-saturation coefficients of w and v_1, respectively (S7 Fig E). Using Eqs. (29)–(32), Eq. (12) can be described by

$$m_1(v_1, w) = \frac{C_{EE}K_w v_1 + C_{ES}K_w K_v + C_{TE}wv_1 + C_{TS}wK_v}{(K_w + w)(K_v + v_1)}, \tag{33}$$

where C_{EE}, C_{ES}, C_{TE}, and C_{TS} are constants.

In *tmm* mutant, we have

$$m_1(v_1, 0) = \frac{C_{ES}K_v + C_{EE}v_1}{K_v + v_1}. \tag{34}$$

In *er erl1 erl2* triple mutant, because the receptor dimers are absent (i.e. $C_{EE} = C_{ES} = C_{TE} = C_{TS} = 0$), we have

$$m_1(v_1, w) = 0. \tag{35}$$

IV. Numerical calculation

Numerical simulations are calculated by Euler's method with a time step $\Delta t = 0.002$ using Eqs. (2)–(10) and (33)–(35), until total time reaches $t = 2000.0$ where patterns no longer change. Hexagonal cells are two-dimensionally arranged with periodic boundary conditions. Initial values of variables are given as their equilibrium with random fluctuation of 1.0 %. In the wild type of Fig 4 and S4 Fig, parameter values

are set to be G = 1.0, A_0 = 0.01, A_1 = 2.0, A_2 = 10.0, B_1 = 10.0, B_2 = 0.1, B_3 = 0.01, B_{1m} = B_{2m} = 20.0, B_g = 1.0, C_0 = 0.0, C_1 =1.0, C_2 =1.2, C_3 = 1.0, D_1 = D_2 = D_3 = D_g = 1.0, K_0 = 1.0, K_1 = K_2 = 0.2, K_3 = 0.1, K_m = 1.0, k_+ = 1.0, k_- = 0.1, d_{u1} = d_{u2} = 0.02, d_{v1} = d_{v2} = 2.0, p = 2, q = 3, K_v = 1.0, K_w = 0.1, C_{EE} = 0.1, C_{ES} = 0.1, C_{TE} = 1.6, and C_{TS} = 0.0. Parameter values of mutants are the same as those of the wild type, except for A_1 = 0.0 in *spch*, A_0 = A_2 = 0.0 in *scrm scrm2*, B_{2m} = 2.0 in *scrm-D*, C_3 = 0.0 in *tmm*, and C_{EE} = C_{ES} = C_{TE} = C_{TE} = 0.0 in *erecta-triple*. Parameter values in S2 Fig are the same as those in Fig 4 except for C_2 = 0.0, C_{EE} = 2.1, C_{ES} = 0.0, C_{TE} = 3.2, and C_{TS} = 0.0. Parameter values in S8, S9, S12 and S13 Figs are the same as those in Fig 4 except for d_v = d_{v1} = d_{v2} = 0.04-2.0 and G = 0.05-5.0 in S8 Fig, p = 1.0-3.0 and q = 1.0-5.0 in S9 Fig, A_1 = 0.2-20.0 and C_3 = 0.0-10.0 in S12 Fig, and initial fluctuation of 1.0-100.0 % in S13 Fig.

References for Supplementary Methods

55. Guseman JM, Lee JS, Bogenschutz NL, Peterson KM, Virata RE, et al. (2010) Dysregulation of cell-to-cell connectivity and stomatal patterning by loss-of-function mutation in Arabidopsis CHORUS (GLUCAN SYNTHASE-LIKE 8). Development 137: 1731-1741.
56. Jewaria PK, Hara T, Tanaka H, Kondo T, Betsuyaku S, et al. (2013) Differential effects of the peptides Stomagen, EPF1 and EPF2 on activation of MAP kinase MPK6 and the SPCH protein level. Plant Cell Physiol 54: 1253-1262.
