# Supplementary figures and images for: Molecular Framework of a Regulatory Circuit Initiating Two-Dimensional Spatial Patterning of Stomatal Lineage
Source: PLoS Genet. 2015 Jul 23;11(7):e1005374. doi: 10.1371/journal.pgen.1005374 (PMC4512730; doi:10.1371/journal.pgen.1005374)

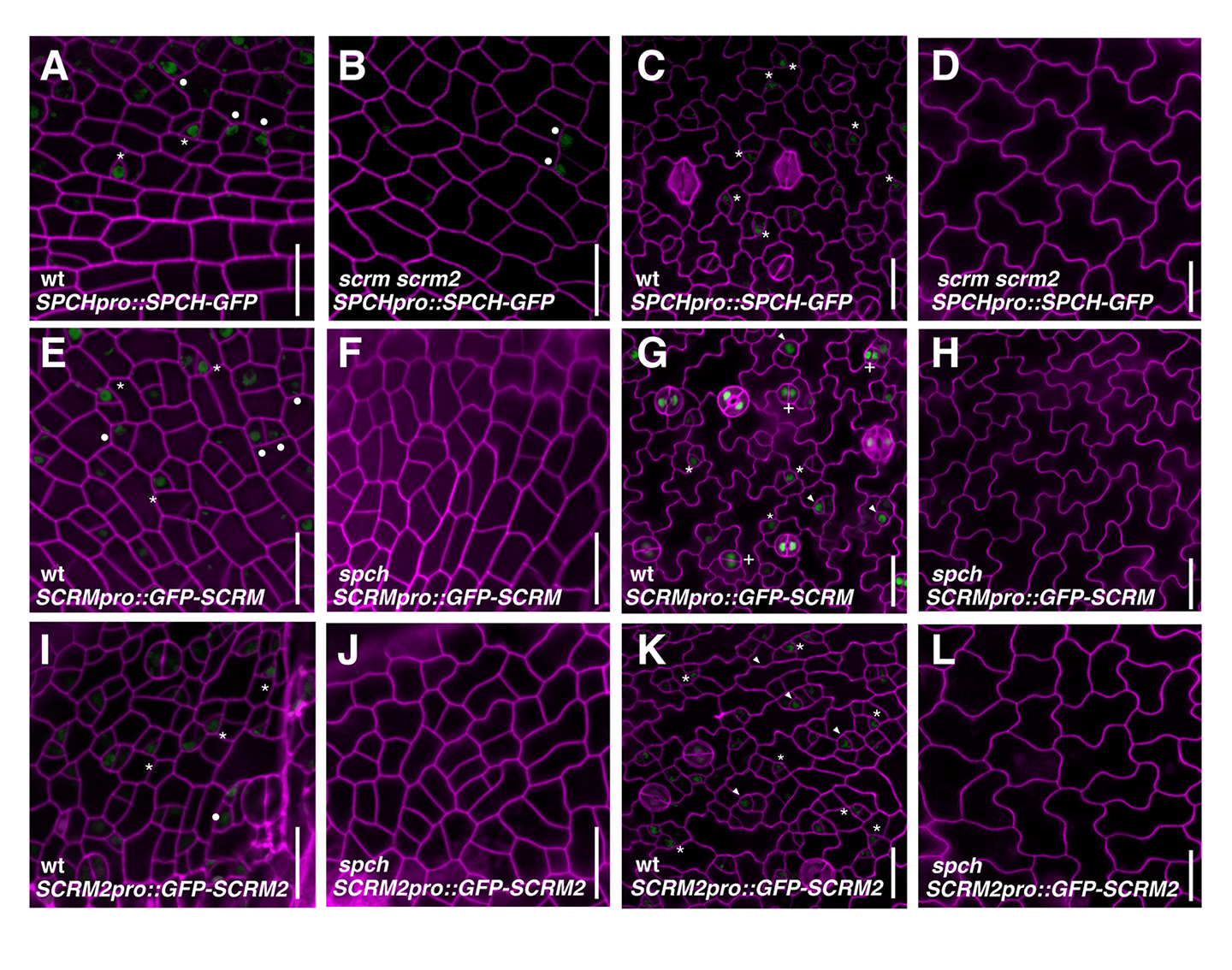

Supplement: S1 Fig — Shown are confocal microscope images of abaxial epidermis from the early protoderm (A, B, E, F, I, J) and developing rosette leaves (C, D, G, H, K, L) of 10-12-day-old Arabidopsis seedlings expressing SPCHpro::SPCH-GFP (A-D), SCRMpro::GFP-SCRM (E-H), and SCRM2pro::GFP-SCRM2 (I-L). These functional GFP-fused constructs are in wild-type (A, C, E, G, I, K) or the opposite knockout mutant backgrounds (SPCHpro::SPCH-GFP in scrm scrm2 [B, D]; SCRMpro::GFP-SCRM and SCRM2pro::GFP-SCRM2 in spch [F, H, J, L]). Note that introduction of SPCHpro::SPCH-GFP into spch, or SCRMpro::GFP-SCRM or SCRM2pro::GFP-SCRM2 into scrm scrm2 rescues the pavement-cell-only mutant phenotypes and therefore cannot be used to investigate the expression of these bHLH proteins in the absence of stomatal-lineage initiation. SPCH-GFP is accumulating in a subset of protodermal cells (A, B; dots) as well in meristemoids (A, C; asterisks). In some instances, SPCH-GFP is detected in dividing protodermal cells in scrm scrm2 despite the absence of stomatal cell lineages (B; dots). In wild type, GFP-SCRM and GFP-SCRM2 are detected in a subset of protodermal cells (E, I; dots), meristemoids (E, G, I, K; asterisks) and guard mother cells (G, K; arrowheads); GFP-SCRM signal remains strong in immature guard cells (g; pluses) and mature guard cells. No GFP-SCRM or GFP-SCRM2 proteins are detected in spch mutant background (F, H, J, L). Scale bars, 20 μm. (TIF) [file pgen.1005374.s005.tif]

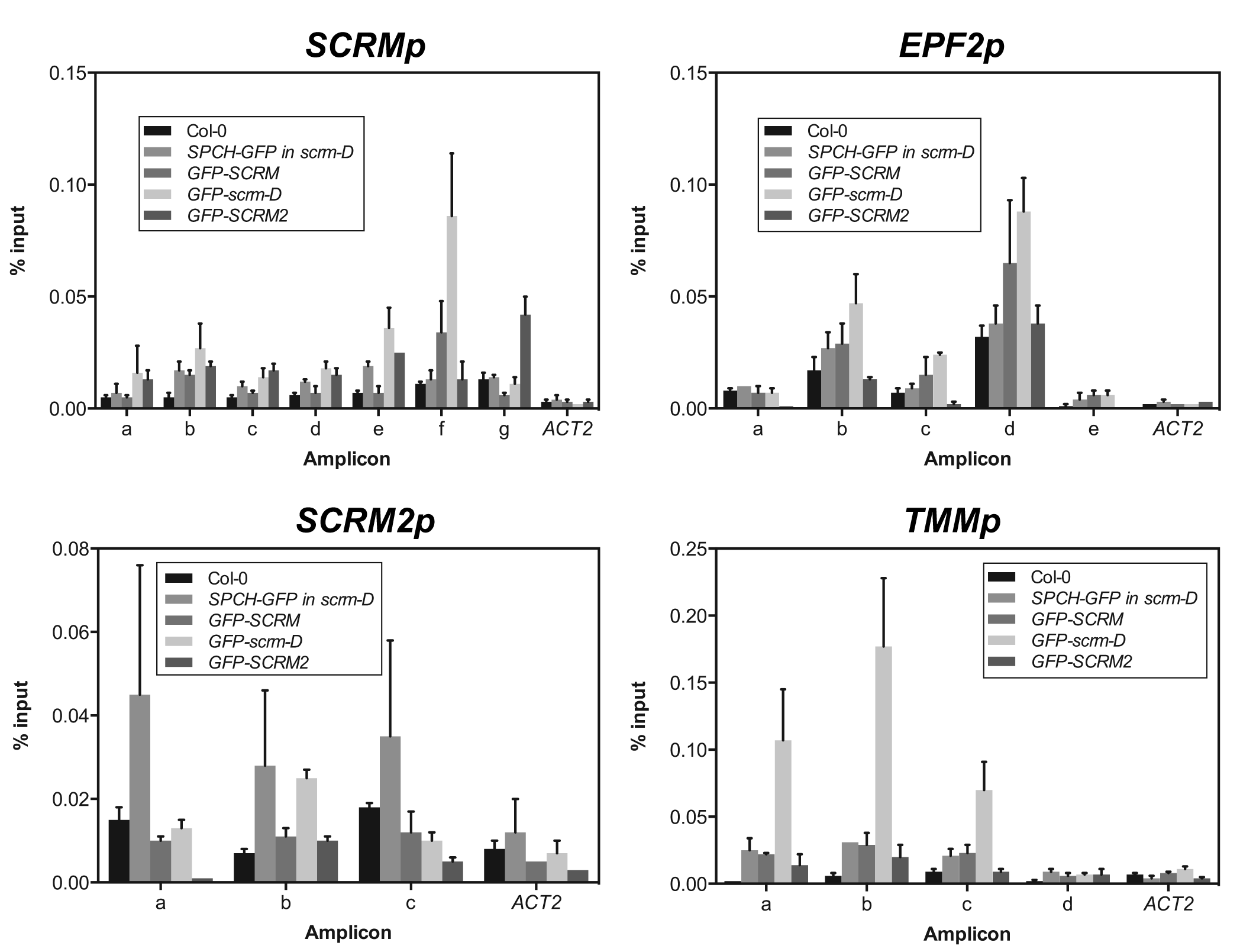

Supplement: S2 Fig — Shown are the same ChIP data as in the main figures (Fig 1D and 1E, Fig 2B, and Fig 3D), but presented as % input. For the location of each amplicon, see main figures. (TIF) [file pgen.1005374.s006.tif]

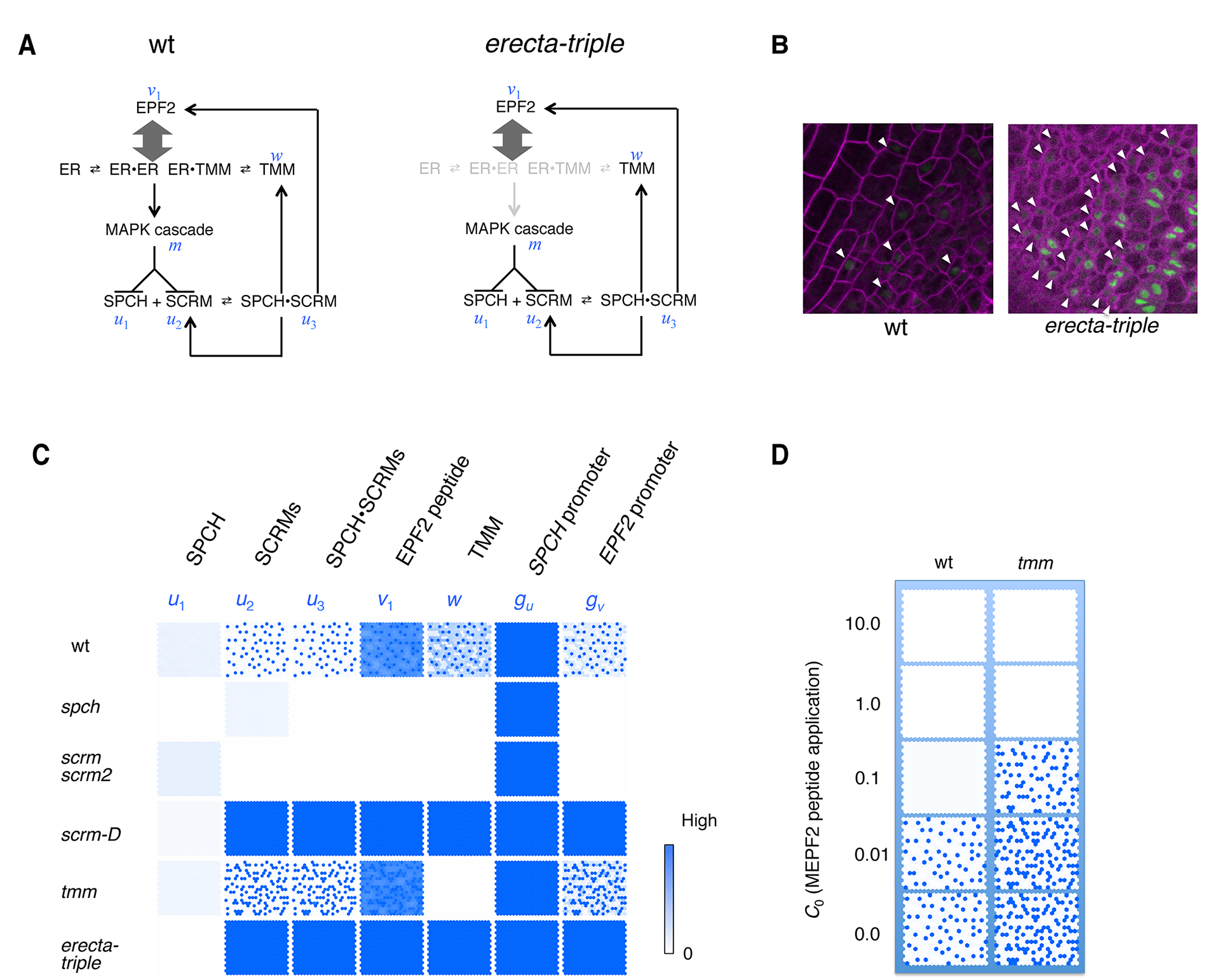

Supplement: S3 Fig — (A) Diagram of regulatory circuit used for the initial modeling. (Left) wild type (wt). (Right) erecta-triple mutant. Arrow designates activation and T-bar designates inhibition. Double arrowheads between EPF2, ER, and TMM indicate combinatorial ligand-receptor associations (see S7 Fig and S1 Text). Concentrations of each components are abbreviated as the following: u 1, SPCH; u 2, SCRM; u 3, SPCH•SCRM heterodimer; v 1, EPF2; w, TMM. m, strength of MAPK-mediated inhibition. (Right) Based on this initial model, MAPK cascade will not be activated in the absence of ERECTA-family, and this results in the entire epidermis adopting stomatal precursor identity. (B) Confocal microscopy of a primary rosette leaf protoderm from one-week-old seedlings of wild-type (left) and erecta-triple mutant (right) expressing SCRMpro::GFP-SCRM. Nuclear accumulation of GFP-SCRM (arrowheads) are spread out in the wild-type protoderm (left), while they are clustered and in erecta-triple mutant (right). Images were taken under the same magnification. (C) Initial spatial patterns of each component in wild type, tmm, and erecta-family triple mutant simulated in silico based on the mathematical models. Each square represents a sheet of protoderm with 400 cells (each cell represented by a hexagon). White cells indicate no expression/accumulation of a given component, while dark blue cells express/accumulate high amounts. In erecta-triple mutant, all epidermal cells become stomatal initials, which is not consistent with the observed phenotype. (D) Sensitivity of wild-type and tmm protoderm to EPF2 application in silico. C 0 designates the concentration of exogenously applied EPF2 (MEPF2) peptide. In this model, tmm is less sensitive to EPF2 than wild type, but the stomatal differentiation can still be inhibited by exogenously applied MEPF2 peptide, which is not consistent with the observed phenotype (see S4 Fig). (TIF) [file pgen.1005374.s007.tif]

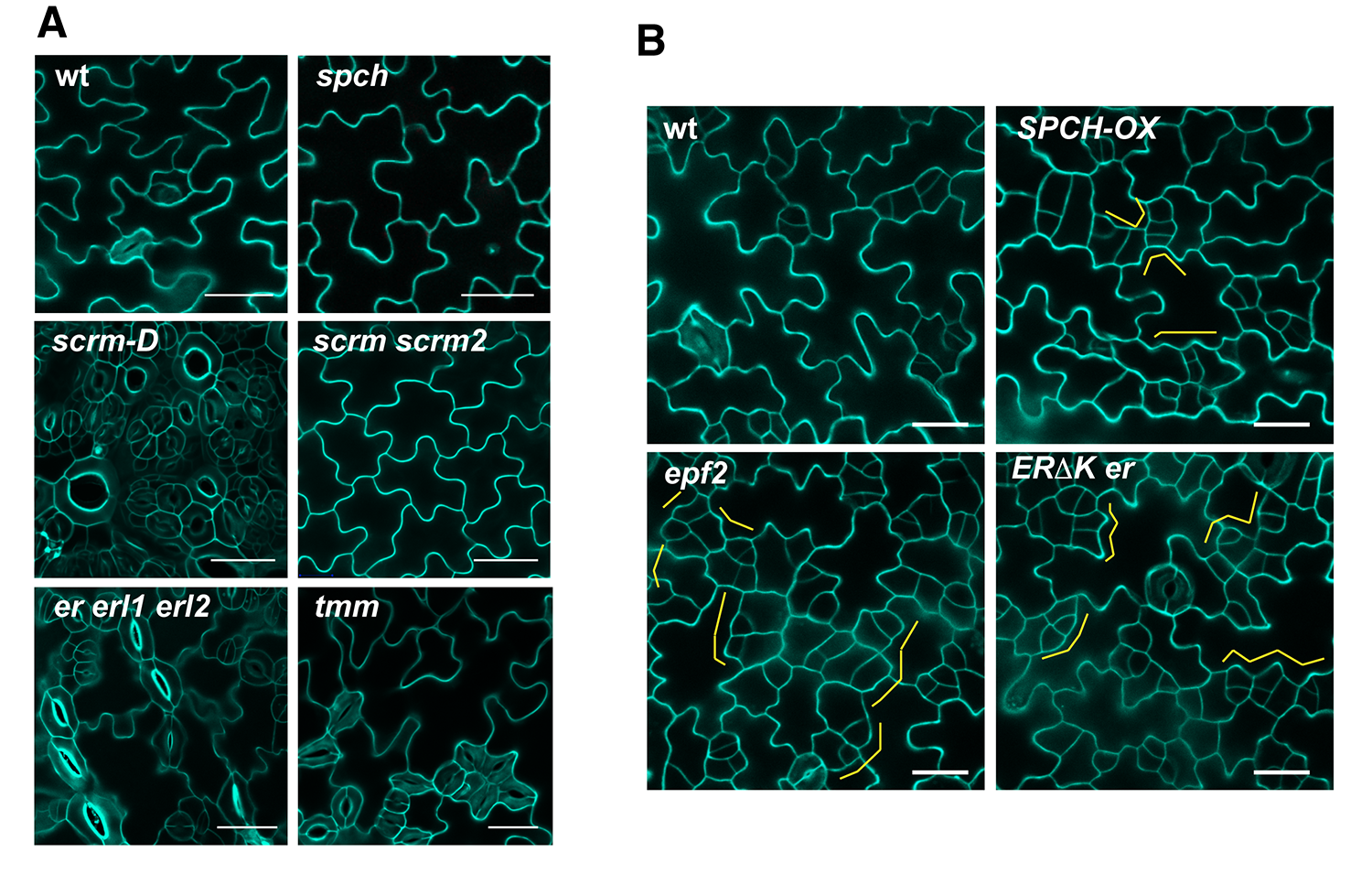

Supplement: S4 Fig — Confocal images of 1-to-2-week-old abaxial cotyledon epidermis. (A) wild type (wt) and other stomatal mutants simulated in this study (spch, scrm-D, scrm scrm2, er erl1 erl2 and tmm). (B) Phenotypic similarity among the activator and inhibitor of stomatal initiation. Shown are confocal images of 5-day-old cotyledon abaxial epidermis. Loss of EPF2 or ERECTA signaling by introduction of a dominant-negative form of ERECTA (ER∆Kinase in erecta) confers a phenotype similar to that of ectopic SPCH overexpression (SPCH-OX). All these plants show excessive entry into stomatal cell lineages (yellow brackets). Scale bar, 20 μm. (TIF) [file pgen.1005374.s008.tif]

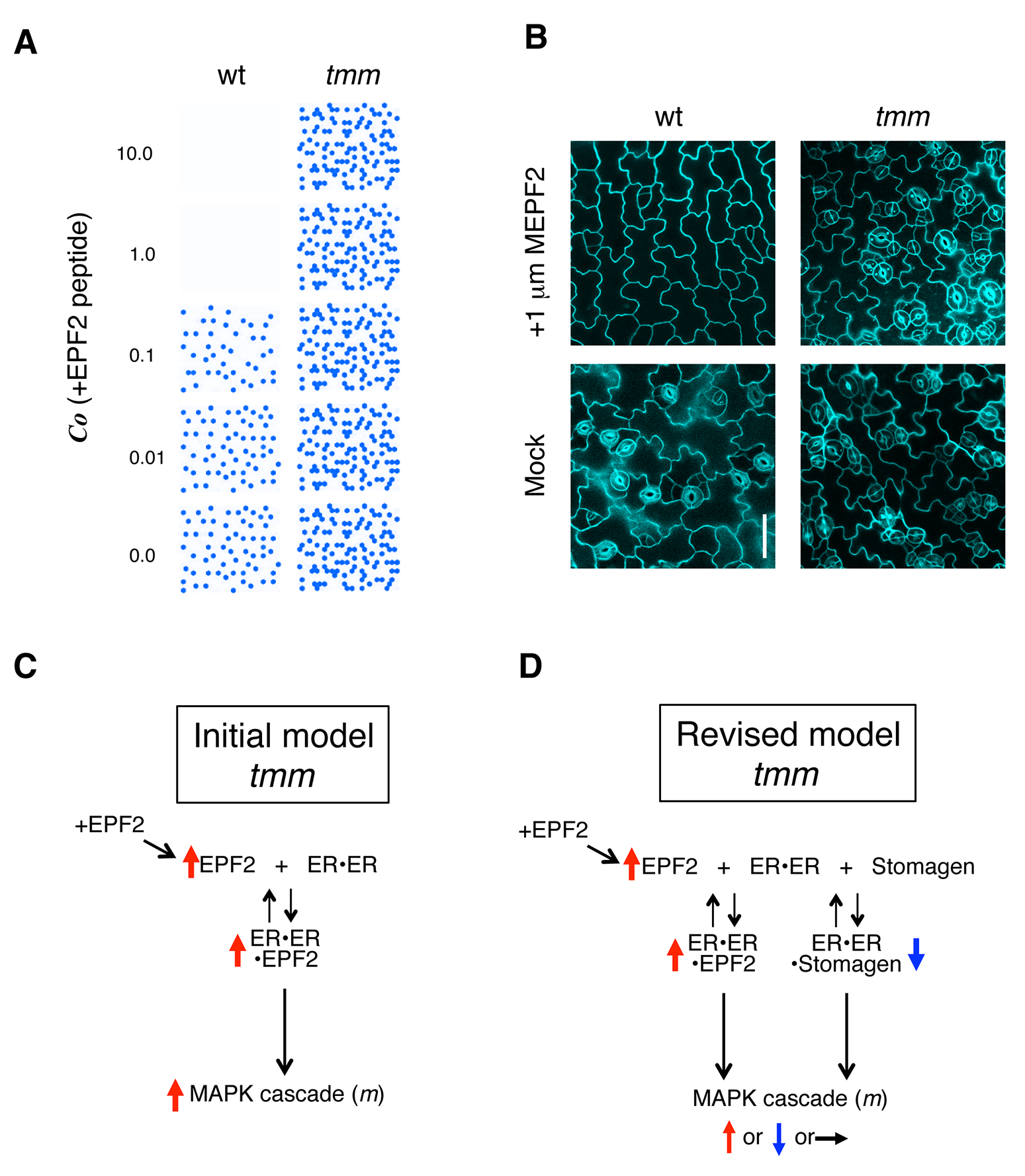

Supplement: S5 Fig — (A) Spatial patterns of stomatal initial cells in wild type and tmm mutants, with increasing amounts of exogenous EPF2 peptide (C 0) simulated in silico. Each square represents a sheet of protoderm with 400 cells (each cell represented by a hexagon). The stomatal initial cells are marked by SPCH•SCRM heterodimers (u 3), with no expression shown as white and maximal expression as dark blue. (B) Application of predicted, mature EPF2 (MEPF2) peptide show no effects on tmm stomatal cluster phenotype. Images of cotyledons from 6-day-old seedlings were taken under the same magnification. Scale bar, 40 μm. (C) Initial model explaining the sensitivity of tmm to EPF2 application. Increased EPF2 triggers inhibitory signals through ERECTA, which activates downstream MAPK cascade and inhibits stomatal differentiation. (D) Revised model explaining insensitivity of tmm to EPF2 application. Here, the presence of a signal (likely Stomagen) that competes with EPF2, balances the activity of ERECTA and maintains the signaling output in the absence of TMM. (TIF) [file pgen.1005374.s009.tif]

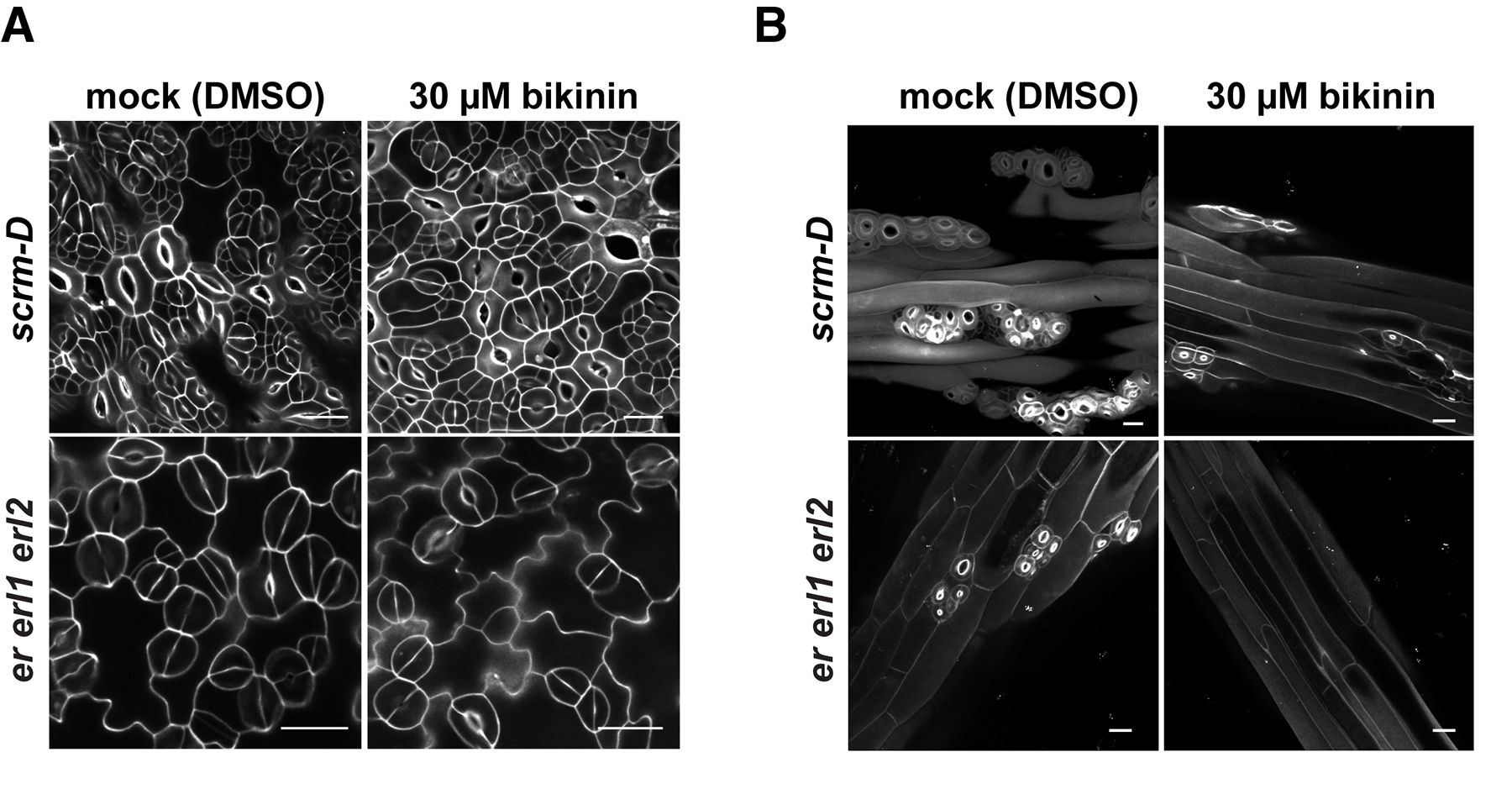

Supplement: S6 Fig — scrm-D and erecta-triple mutant (er erl1 erl2) seedlings were germinated and grown for 8 days on 30 μM bikinin (right panels) or without bikinin (left panels). (A) Representative confocal microscopy images of cotyledon abaxial epidermis. As reported previously [22], bikinin treatment confers no effects on scrm-D stomata-only phenotypes, while stomatal clustering phenotype of er erl1 erl2 gets alleviated. (B) Representative confocal microscopy images of hypocotyl epidermis. Bikinin treatment reduces stomatal clusters in scrm-D and, surprisingly, completely suppress stomatal differentiation in er erl1 erl2. Scale bar, 20 μm. (TIF) [file pgen.1005374.s010.tif]

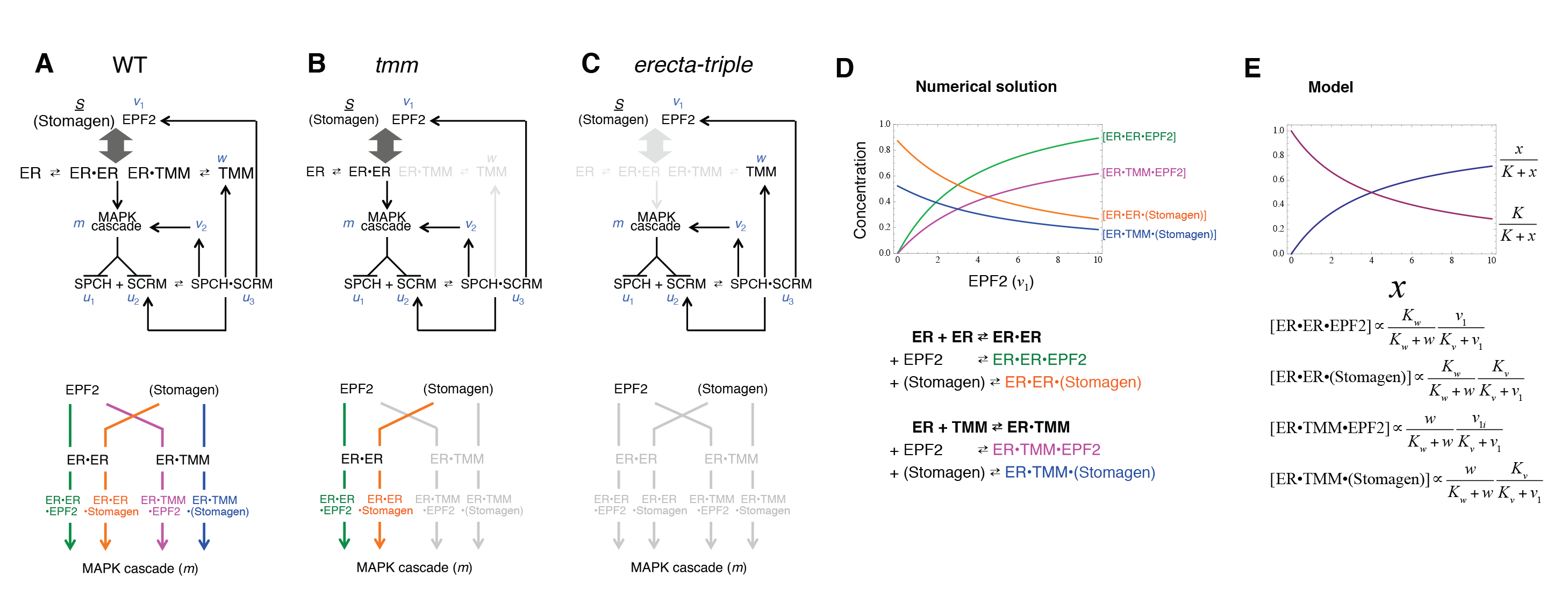

Supplement: S7 Fig — (A-C) Top: Shown are regulatory circuit diagrams of wild type (WT: A), tmm (B), and erecta (er)-family triple (C) mutant used for our mathematical modeling presented in Fig 4 and S4 Fig. Concentrations of each component are abbreviated as the following: u 1, SPCH; u 2, SCRM; u 3, SPCH•SCRM heterodimer; v 1, EPF2; w, TMM; v 2, EPF2-independent hypothetical component. m, strength of MAPK-mediated inhibition. S, a component that competes for receptor pools, most likely Stomagen. Bottom: Available ligand-receptor pools and additional components that activate MAPK to inhibit stomatal initials in each scenario. (D) Example of numerical solution of the ligand-receptor complex concentrations for Eqs. (19)–(28) in S1 Text with parameter condition of k 1 = k 2 = 1.0, k 3 = k 4 = k 5 = k 6 = 2.0, E 0 = 3.5, S 0 = 3.0, and w = 1.0. (E) Increasing and decreasing changes in concentrations of ligand-receptor complexes (D) are approximated by x/(K + x) and K/(K + x), respectively, in our model. (TIF) [file pgen.1005374.s011.tif]

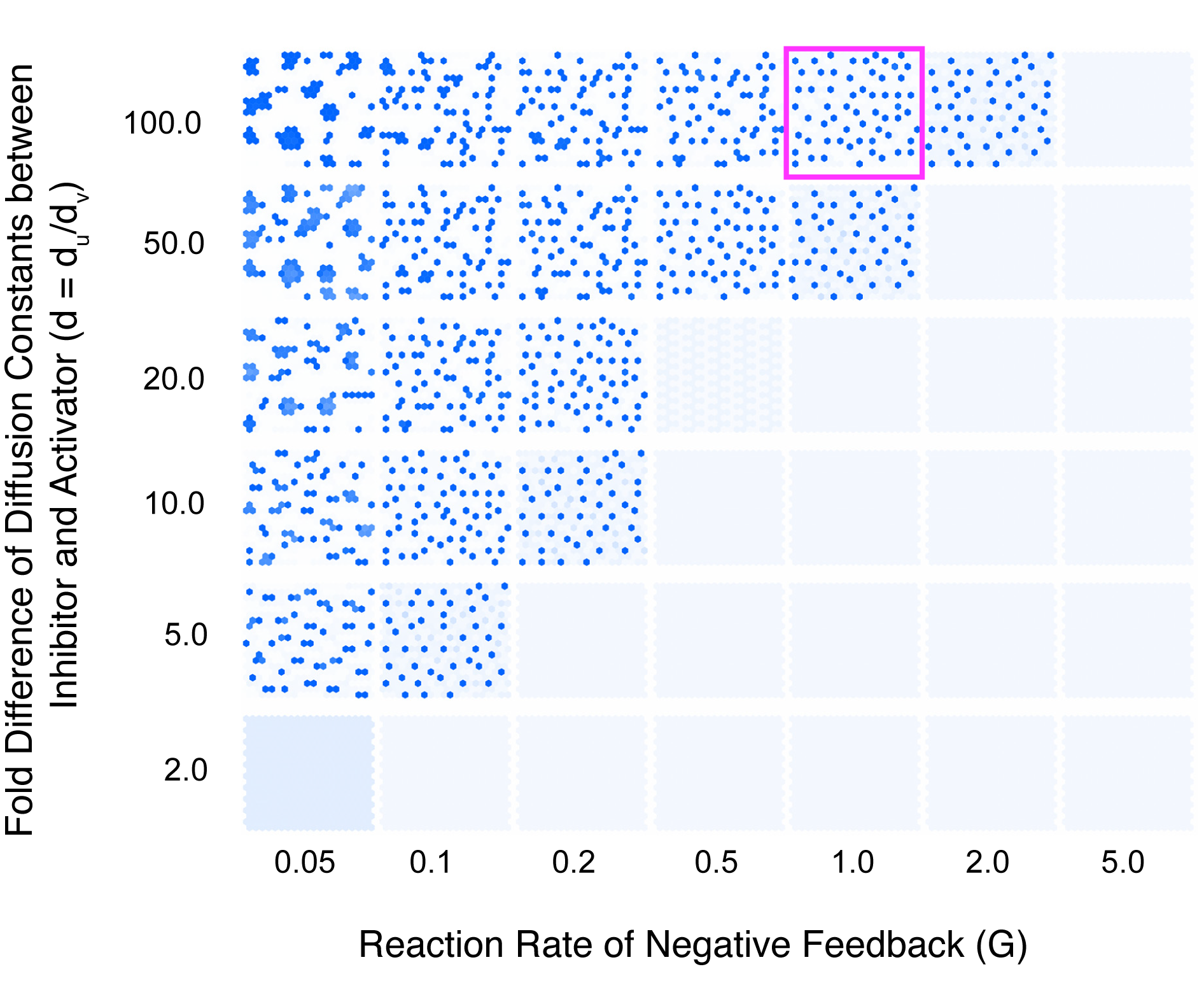

Supplement: S8 Fig — Shown are simulations of stomatal lineage initiation patterns calculated with a range (2.0–100.0) of the ratio of diffusion constants (d = d v/d u) of inhibitors (d v = d v1 = d v2) over activators (d u = d u1 = d u2 = 0.02) in a function of a range (0.05–5.0) of parameter G. G is a reaction rate coefficient of the negative feedback loop (see Eqs. (6)–(8) in S1 Text). As a value of G decreases, the optimal value for d for proper stomatal patterning decreases. Highlighted in pink rectangle is our standard simulation condition of G = 1.0 and d = 100.0 (see S1 Text). Blue, cells accumulating SPCH•SCRMs (u 3); White, cells with no expression/accumulation. (TIF) [file pgen.1005374.s012.tif]

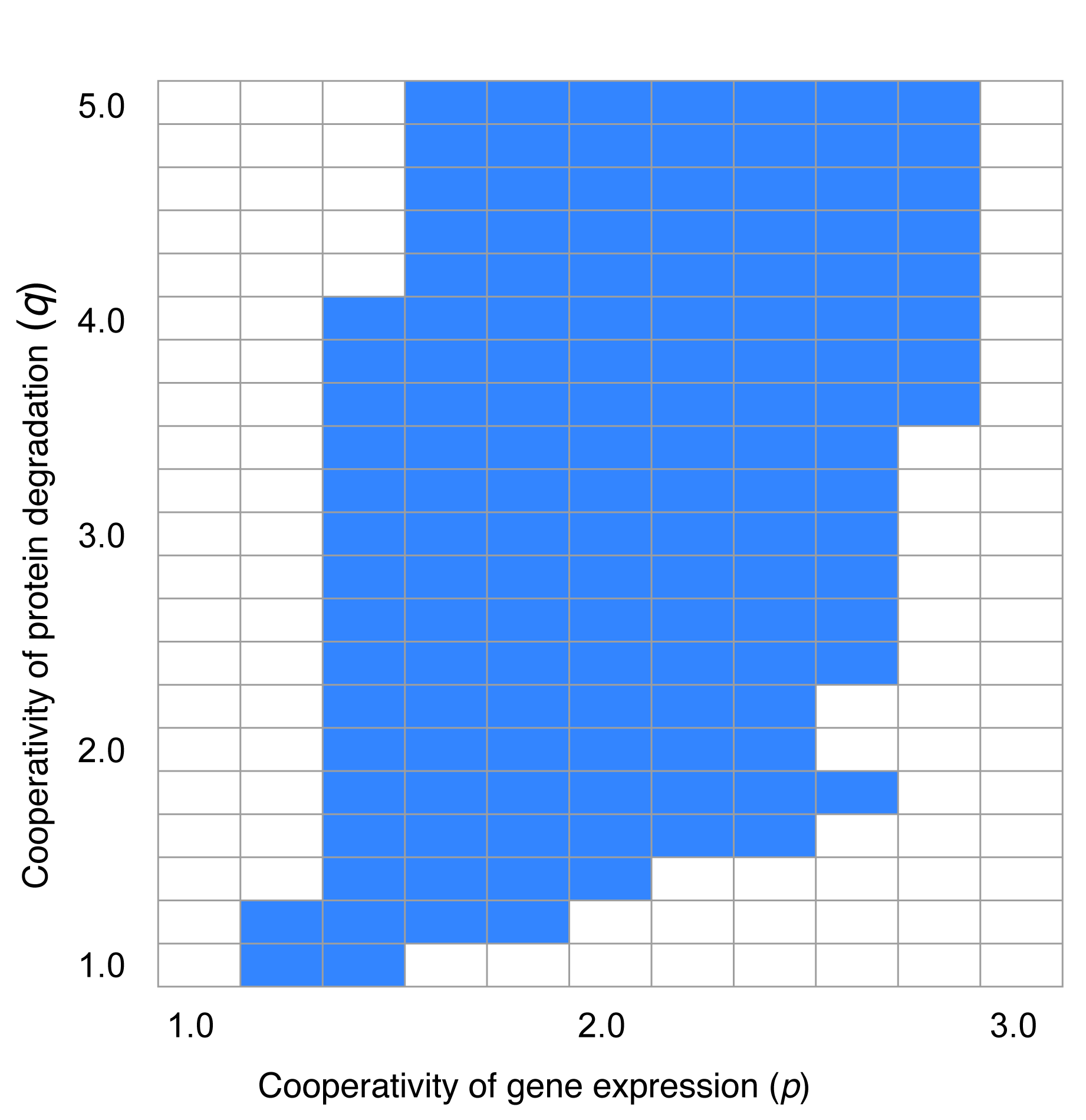

Supplement: S9 Fig — Shown are simulations of stomatal lineage initiation patterns when Hill coefficients for parameters p (cooperativity of SCRMs, EPF2 and TMM gene expression by SPCH•SCRMs) and q (cooperativity of SPCH and SCRM protein degradation) are altered. Here simulations were done with p = 1.0–3.0 and q = 1.0–5.0. See Eqs. (3)–(8) for parameters p and q (S1 Text). Values for cooperativity p ranging between 1.4 and 2.6 (when q = 3.0) are required for spatial patterning of stomatal initials. Conditions that create any stomatal-lineage initials are highlighted in blue. (TIF) [file pgen.1005374.s013.tif]

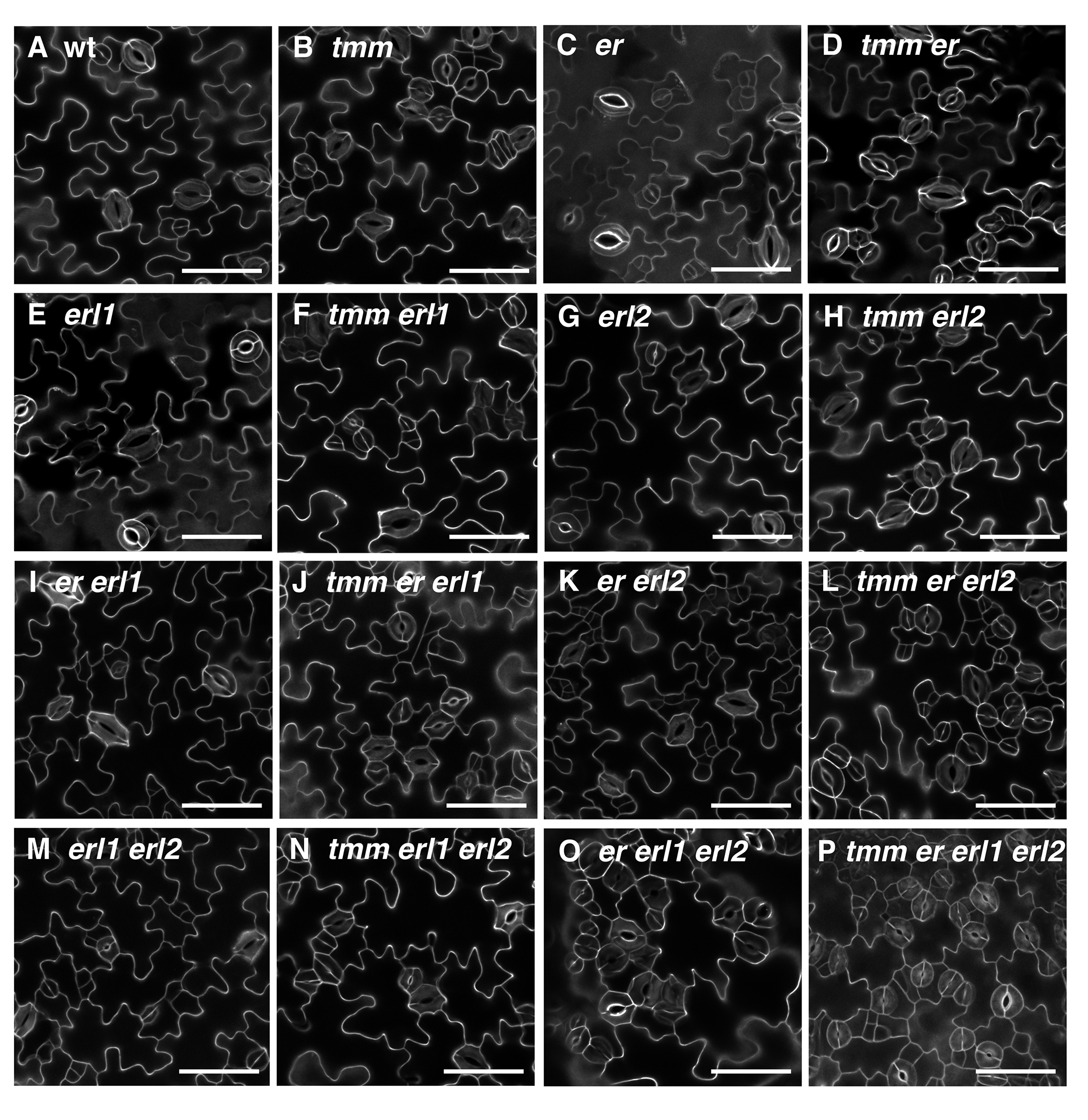

Supplement: S10 Fig — Shown are representative confocal microscopy images of abaxial cotyledon epidermis from seven-day-old seedlings of: (A) wild type (wt), (B) tmm, (C) er, (D) tmm er, (E) erl1, (F) tmm erl1, (G) erl2, (H) tmm erl2, (I) er erl1, (J) tmm er erl1, (K) er erl2; (L) tmm er erl2; (M) erl1 erl2; (N) tmm erl1 erl2; (O) er erl1 erl2; (P) tmm er erl1 erl2. The cotyledons from any combination of er-family higher order mutants with additional tmm mutation exhibit stomatal clusters. Scale bars, 50 μm. (TIF) [file pgen.1005374.s014.tif]

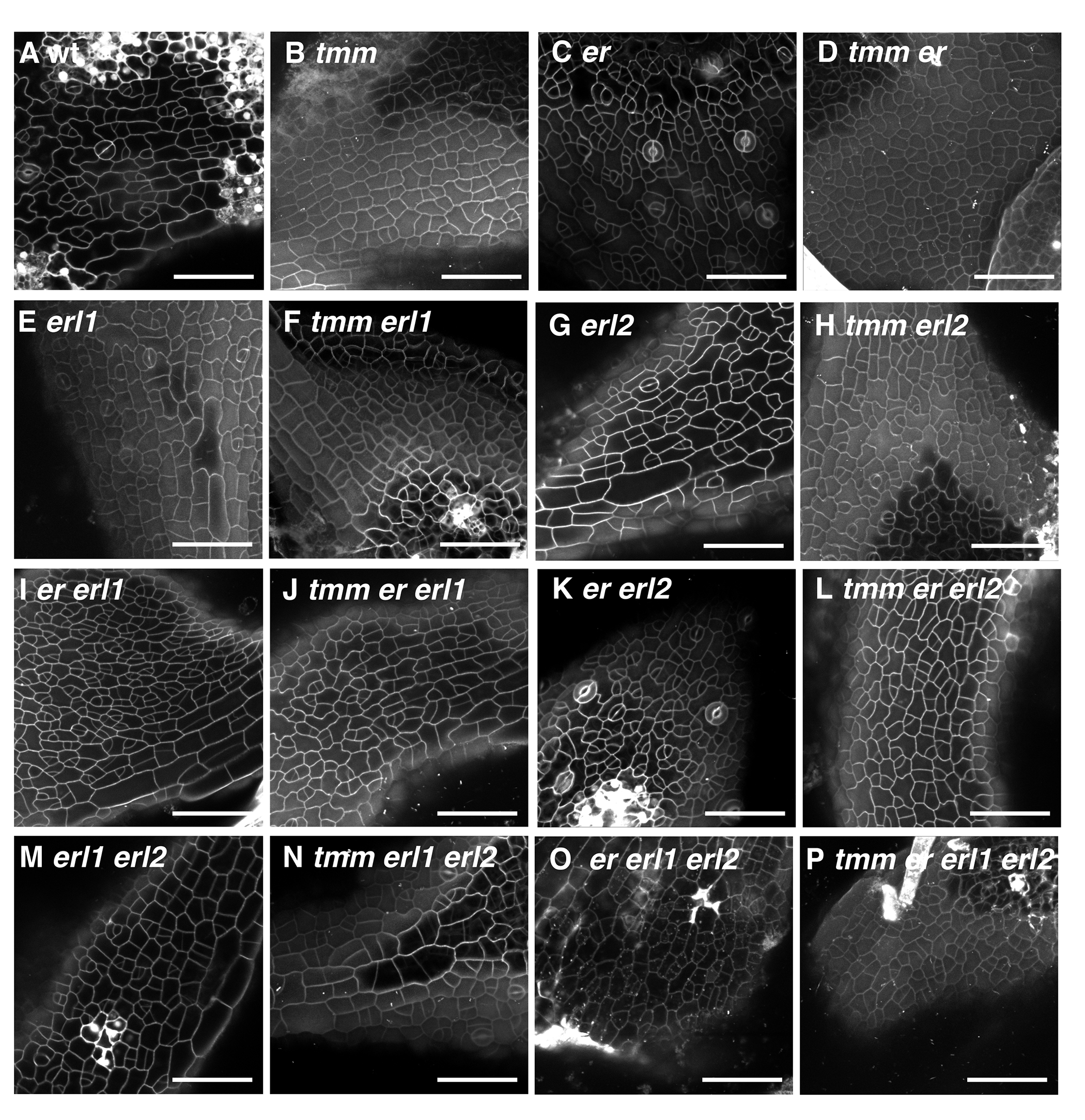

Supplement: S11 Fig — Shown are representative confocal microscopy images of abaxial rosette leaf protoderm from 7 or 8-day-old seedlings of: (A) wild type (wt), (B) tmm, (C) er, (D) tmm er, (E) erl1, (F) tmm erl1, (G) erl2, (H) tmm erl2, (I) er erl1, (J) tmm er erl1, (K) er erl2; (L) tmm er erl2; (M) erl1 erl2; (N) tmm erl1 erl2; (O) er erl1 erl2; (P) tmm er erl1 erl2. The additional tmm mutation appears to increase meristemoids in er-family higher order mutants. Scale bars, 50 μm. (TIF) [file pgen.1005374.s015.tif]

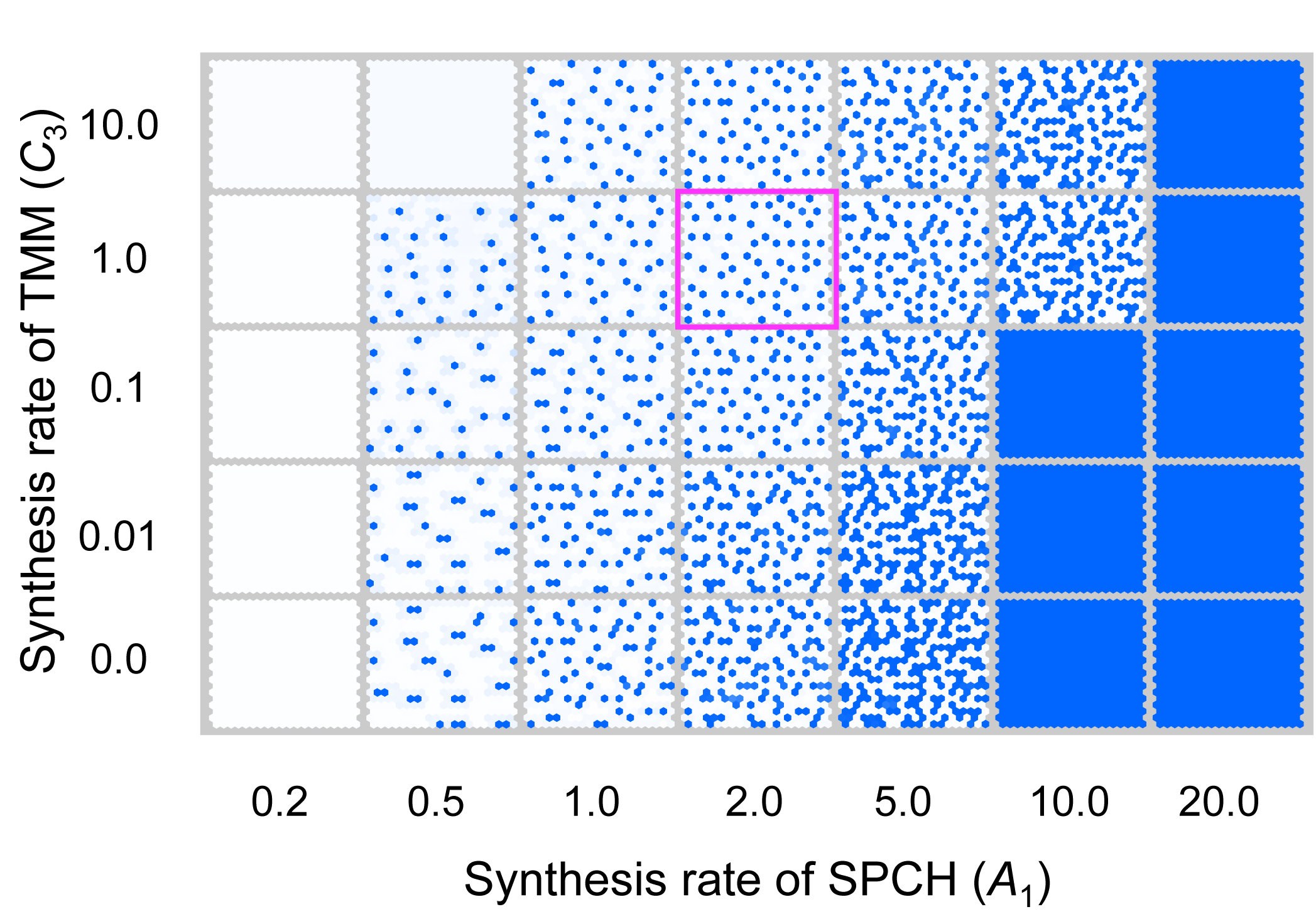

Supplement: S12 Fig — Shown are simulations of stomatal lineage initiation patterns in the range of a synthesis rate of TMM (C 3, 0.0–10.0) and that of SPCH (A 1, 0.2–20.0). See Eqs. (7) and (3) for C 3 and A 1, respectively (S1 Text). Synthesis rate of SPCH greatly influences distribution and patterning of stomatal initial cells, whereas synthesis rate of TMM has a modest role in enforcing spacing. Blue, high levels of SPCH•SCRMs (u 3); White, cells with no expression/accumulation. Highlighted in pink rectangle is our standard simulation condition of A 1 = 2.0 and C 3 = 1.0. (TIF) [file pgen.1005374.s016.tif]

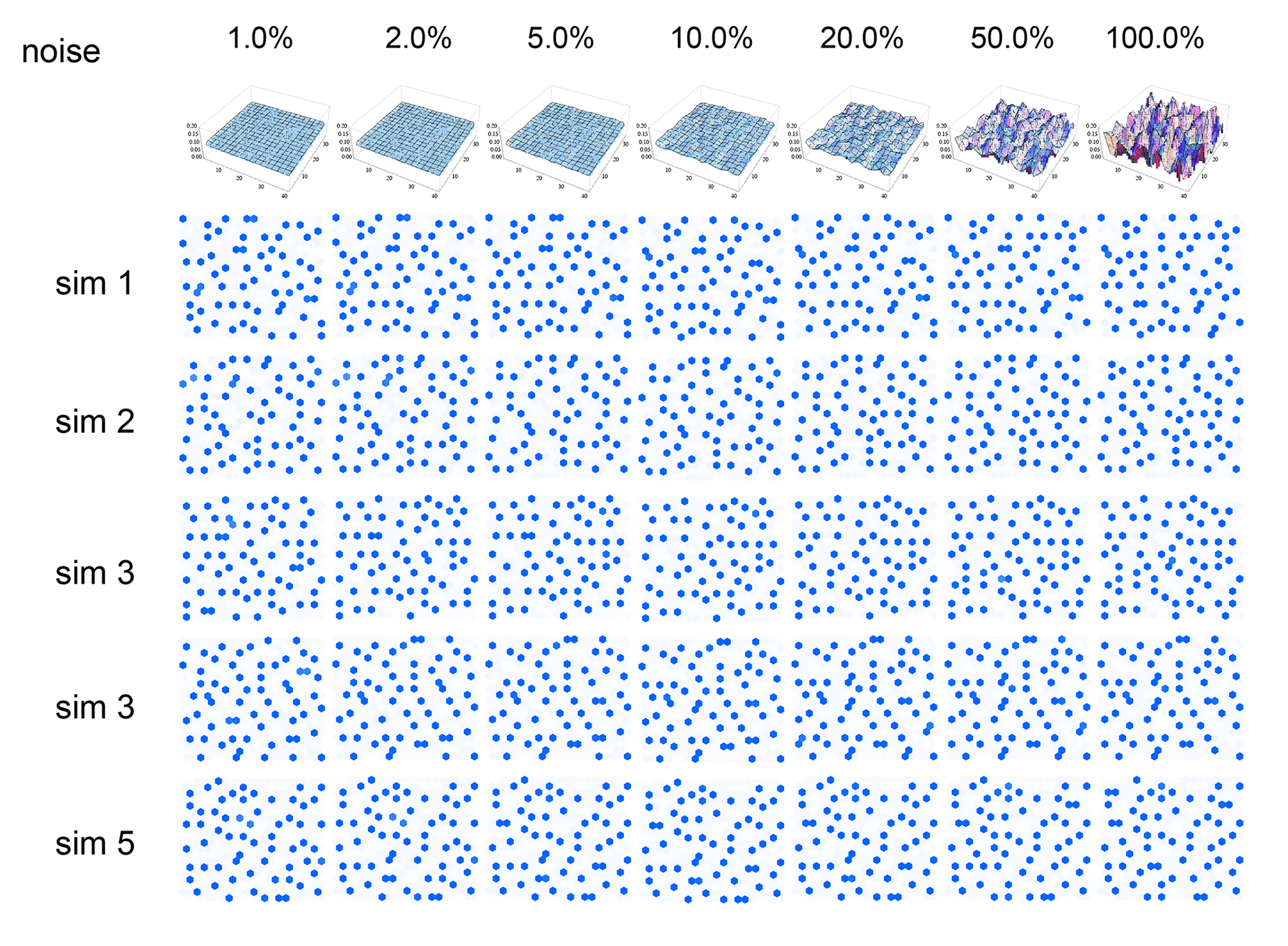

Supplement: S13 Fig — Shown are five independent simulations with random noise being introduced to the initial values of all components (percentages of noise are indicated above each column). Shown in the top row are representative of SPCH•SCRMs (u 3) initial distribution in two-dimensional space (20 x 20 hexagons per each condition) upon introduction of respective noise. Shown in blue are cells expressing SPCH•SCRMs; white—no expression. (TIF) [file pgen.1005374.s017.tif]
